# Relative Contributions of Specific Activity Histories and Spontaneous Processes to Size Remodeling of Glutamatergic Synapses

**DOI:** 10.1371/journal.pbio.1002572

**Published:** 2016-10-24

**Authors:** Roman Dvorkin, Noam E. Ziv

**Affiliations:** 1 Technion Faculty of Medicine, Network Biology Research Laboratories, Lorry Lokey Center for Life Sciences and Engineering, Technion, Haifa, Israel; 2 Rappaport Family Institute for Research in the Medical Sciences, Haifa, Israel; Max Planck Institute for Brain Research, GERMANY

## Abstract

The idea that synaptic properties are defined by specific pre- and postsynaptic activity histories is one of the oldest and most influential tenets of contemporary neuroscience. Recent studies also indicate, however, that synaptic properties often change spontaneously, even in the absence of specific activity patterns or any activity whatsoever. What, then, are the relative contributions of activity history-dependent and activity history-independent processes to changes synapses undergo? To compare the relative contributions of these processes, we imaged, in spontaneously active networks of cortical neurons, glutamatergic synapses formed between the same axons and neurons or dendrites under the assumption that their similar activity histories should result in similar size changes over timescales of days. The size covariance of such commonly innervated (CI) synapses was then compared to that of synapses formed by different axons (non-CI synapses) that differed in their activity histories. We found that the size covariance of CI synapses was greater than that of non-CI synapses; yet overall size covariance of CI synapses was rather modest. Moreover, momentary and time-averaged sizes of CI synapses correlated rather poorly, in perfect agreement with published electron microscopy-based measurements of mouse cortex synapses. A conservative estimate suggested that ~40% of the observed size remodeling was attributable to specific activity histories, whereas ~10% and ~50% were attributable to cell-wide and spontaneous, synapse-autonomous processes, respectively. These findings demonstrate that histories of naturally occurring activity patterns can direct glutamatergic synapse remodeling but also suggest that the contributions of spontaneous, possibly stochastic, processes are at least as great.

## Abstract 2

Contrary to expectations, specific activity histories account for less than half of the remodeling exhibited by individual glutamatergic synapses in spontaneously active networks of cortical neurons.

## Author Summary

The modification of synaptic connections by specific activity histories (a phenomenon known as synaptic plasticity) is widely believed to represent a major substrate of processes collectively referred to as learning and memory. Recent studies indicate, however, that synapses also change spontaneously, even in the absence of specific activity histories—or, for that matter, any activity whatsoever. This raises a fundamental question: how do changes directed by specific activity histories quantitatively compare to spontaneous changes in synaptic properties? Put differently—what is the “signal-to-noise ratio” of synaptic plasticity at individual synapses? To address this question we followed—over several days—pairs of synapses formed between the same neurons under the assumption that their common activity histories should drive similar changes in their sizes. Indeed, sizes of such synapses tended to change in a correlated manner; yet the extent of this correlation was surprisingly modest, accounting for less than half of the changes that such synapses exhibited. Moreover, sizes of synapses with apparently common activity histories tended to be quite different. Our findings thus indicate that the “signal-to-noise ratio” of synapse remodeling might be rather poor, on the order of 1:1 or less.

## Introduction

Activity-induced modification of synaptic connections (synaptic plasticity) is widely believed to represent a major mechanism for modifying the functional properties of neuronal networks. Indeed, overwhelming experimental evidence supports the idea that synaptic properties are affected by the history of their activation. What is less established and often ignored is the "flip side" of synaptic plasticity: that is, the implicit supposition that synapses, when not driven to change their characteristics, will retain these over time. This assumption would seem to be an essential complement of the synaptic plasticity concept; without it, spontaneous changes occurring independently of physiologically relevant input would cause spurious changes in network function or undo physiologically relevant ones.

The validity of this assumption has been called into question by recent studies, in which sizes and contents of individual synapses—both excitatory and inhibitory—were observed to fluctuate considerably over timescales of hours and days (e.g., [1–17]); notably, such fluctuations persisted even in the absence of specific activity patterns or any activity at all (e.g., [5,6,9,12,17]). Finally, it was shown that these fluctuations could be described remarkably well by statistical processes that are essentially stochastic [5,6,8,16,17]. Given the emerging view of the synapse as a complex assembly of dynamical components [1,2], the presence of such fluctuations might not be very surprising. Nevertheless, they would seem to imply that synaptic tenacity, which we define as the capacity of individual synapses to maintain their properties over behaviorally relevant time scales [6,9,11,17], is inherently limited, and that synapses exhibit a non-negligible degree of spontaneous size remodeling.

Although these conclusions were derived mainly from studies in reduced systems (cell and organotypic cultures), they are not limited to these settings [4,8,14,15]. Thus, for example, it has recently been shown that synapse size fluctuations in the cerebral cortex of adult mice are at least as large as those observed in culture ([15]; see also [4]); in fact, the degree of such size fluctuations is comparable to the magnitude of size changes induced by experimental stimulation paradigms that induce long-term potentiation (e.g., [18,19]). Thus, when considering changes in synaptic sizes, it remains to be asked what the relative contributions of specific activity histories to such changes are and how these compare to size changes driven by other, possibly stochastic, processes.

In the rodent cerebral cortex, two neurons are often connected by more than one excitatory synapse (reviewed in [20]). This situation provides an excellent opportunity to examine the relative contributions of specific activity histories to changes in synaptic sizes and then compare these to the contributions of other processes. This claim is based on the reasonable premise that, to a first approximation, all synapses connecting two specific neurons (commonly innervated [CI] synapses) will have similar activation histories when these are integrated over many days [21,22]. Assuming that changes in synaptic properties are driven primarily by activation histories, changes in the sizes of such CI synapses might be expected to co-vary significantly. In contrast, synapses formed on the same neuron or dendrite by two different upstream neurons (non-commonly innervated [non-CI] synapses), would have somewhat different activation histories, and thus their sizes would not be expected to co-vary to the same degree. Moreover, the remodeling covariance would be expected to be even greater for nearby synapses formed between the same axonal and dendritic segments, as regional differences in axonal/dendritic properties would minimally affect activity histories and their biological consequences. Finally, this approach provides an opportunity to examine how synaptic sizes are affected by more natural activation histories, spanning hours and days, as compared to the brief and rather artificial stimulation paradigms typically used in experimental settings (reviewed in [23]).

In the current study we measured and compared the remodeling of CI and non-CI synapses in monolithic and modular networks of cortical neurons in primary culture by using genetically encoded fluorescent reporters combined with multielectrode array (MEA) recordings, automated confocal microscopy, and pharmacological manipulations. Although cortical networks in culture differ in many ways from their in vivo counterparts, in the current context, they are advantageous in the sense that they provide a generic, isolated, and well-controlled system for studying the net effects of activation histories, free from potential confounds inherent to in vivo settings such as behavioral states, stress, neuromodulatory input, and circadian rhythms. Moreover, as shown below, this system allows for excellent long-term and continuous monitoring of synaptic sizes, the presynaptic origins of individual synapses, and experimental differentiation of activation histories. Our findings are presented next.

## Results

### Rationale and Experimental Approach

**Fig 1:**
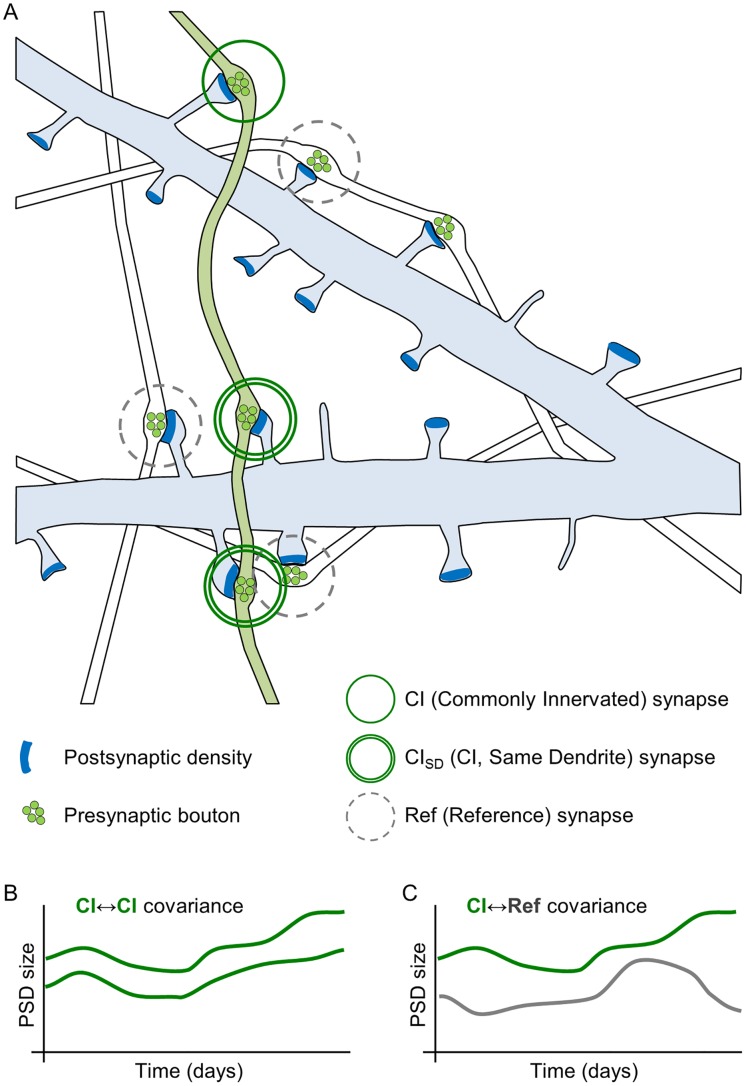
Rationale and experimental design. (A) The experiments described here are based on the assumption that synapses formed between the same axon (green) and the same neuron (blue) will have similar activity histories, particularly when such synapses are located on the same dendrite. Such synapses are referred to as CI and CI_SD_, respectively. For each CI synapse, a Ref synapse is selected that is connected to a different upstream neuron (white). (B) Given their similar activity histories, changes in the sizes of synapses belonging to the same CI pair might be expected to co-vary more than the sizes of two synapses innervated by different axons, i.e., a CI synapse and a Ref synapse (C).

The rationale of the experiments described below is depicted in Fig 1A. In this scheme, a single postsynaptic neuron is innervated by multiple axons belonging to different “upstream” excitatory neurons. A subset of synapses formed on this postsynaptic cell represents connections formed with a particular upstream axon, and these are hereafter referred to as CI synapses. Some CI synapses are located on the same dendrite, and these are hereafter referred to as Commonly Innervated Same Dendrite (CI_SD_) synapses. For each CI synapse, a nearby synapse is selected, which represents a connection between the postsynaptic neuron and another axon. These are hereafter referred to as reference (Ref) synapses. As explained above, it might be expected that CI synapses will have very similar activation histories (even more so, perhaps, for CI_SD_ synapses). If activation history is the major force that drives changes in synaptic size, then CI synapses should change in a similar manner, resulting in a strong covariance of their sizes over time (as illustrated schematically in Fig 1B). Similarly, given that CI and Ref synapses are activated by different upstream neurons and assuming that the activity histories of these neurons differ significantly (a matter we will return to later), sizes of CI and Ref synapses (non-CI synapses) would not be expected to co-vary to the same extent (Fig 1C), with the residual covariance mainly representing the combined contributions of (postsynaptic) neuron- (or dendrite-) wide, non-synapse-specific processes. The overall goals were therefore to (1) quantify the covariance of CI synapses, (2) compare it to the covariance of non-CI synapses, and (3) use these data to estimate the specific contributions of particular activity histories to the remodeling of glutamatergic synapses.

The experiments were carried out in a system based on networks of rat cortical neurons growing on thin glass MEA substrates, automated confocal microscopy, and genetically encoded fluorescent reporters. This system, which we have previously used to explore relationships between activity and remodeling of excitatory [6,16,24] and inhibitory synapses [17], allows for chronic recordings of network activity from up to 59 electrodes while simultaneously imaging synapses by automated confocal microscopy for many days, even weeks. For these experiments, we used cortical networks maintained in culture for 18–21 d, as at this time, synaptogenesis is mostly complete and synapses are relatively mature. To estimate changes in synaptic sizes, we expressed fluorescently tagged variants of the postsynaptic density (PSD) protein PSD-95 (/Dlg4/SAP90) and followed changes in its fluorescence at individual synapses. PSD-95 is a core postsynaptic scaffolding protein of glutamatergic synapses that is thought to control the number of glutamate receptors at the postsynaptic membrane through direct and indirect interactions (reviewed in [25]; see also [26]). Importantly, a recent in vivo correlative light and electron microscopy study [15] demonstrated excellent correlations between tagged PSD-95 fluorescence and PSD area when these are measured for the same synapses, and thus fluorescently tagged PSD-95 can be used to record changes in PSD area and, by extension, in synaptic size. To locate CI synapses, we expressed spectrally separable fluorescently tagged variants of presynaptic molecules, namely SV2 (a conserved, highly specific synaptic vesicle integral membrane protein; [27,28]) or Synapsin I (a synaptic vesicle-associated phosphoprotein [29]; experiments described later on). Expression of all fluorescent reporters was carried out using lentiviral vectors, resulting in minimal overexpression levels of exogenous proteins and very sparse labeling of individual neurons. In spite of the sparse labeling, postsynaptic sites (labeled with fluorescently tagged PSD-95) juxtaposed against fluorescent presynaptic sites (labeled with fluorescently tagged SV2 or Synapsin I) were often observed. Careful examination then allowed us to locate pairs (and sometimes triplets or more) of CI synapses, that is, postsynaptic sites connected to the same axon. As axonal shafts were often barely discernable, the selection of CI synapses for subsequent analyses was limited to short axonal stretches for which a common axonal origin could be determined unambiguously (see Materials and Methods for further details). Fluorescently tagged CI synapses were then followed over time to verify that presynaptic and postsynaptic compartments remained juxtaposed at all time points. A Ref synapse was then chosen near each synapse connected to the common axon, and tagged PSD-95 fluorescence was measured at all synapses—CI and Ref alike—at each time point for the duration of the experiments (all measurements were made in maximum intensity projections of all sections). To minimize the potential effects of measurement noise, fluorescence measures of each synapse were first smoothed with a 2.5- to 3-h low-pass filter [16]. The fluorescence covariance of all CI and non-CI synapse pairs was then calculated using Pearson’s correlation (a linear measure) as well as Spearman’s rank correlation (a measure that quantifies monotonic, but not necessarily linear, relationships between two variables).

### Covariance of CI and non-CI Synapses in Monolithic Networks

**Fig 2:**
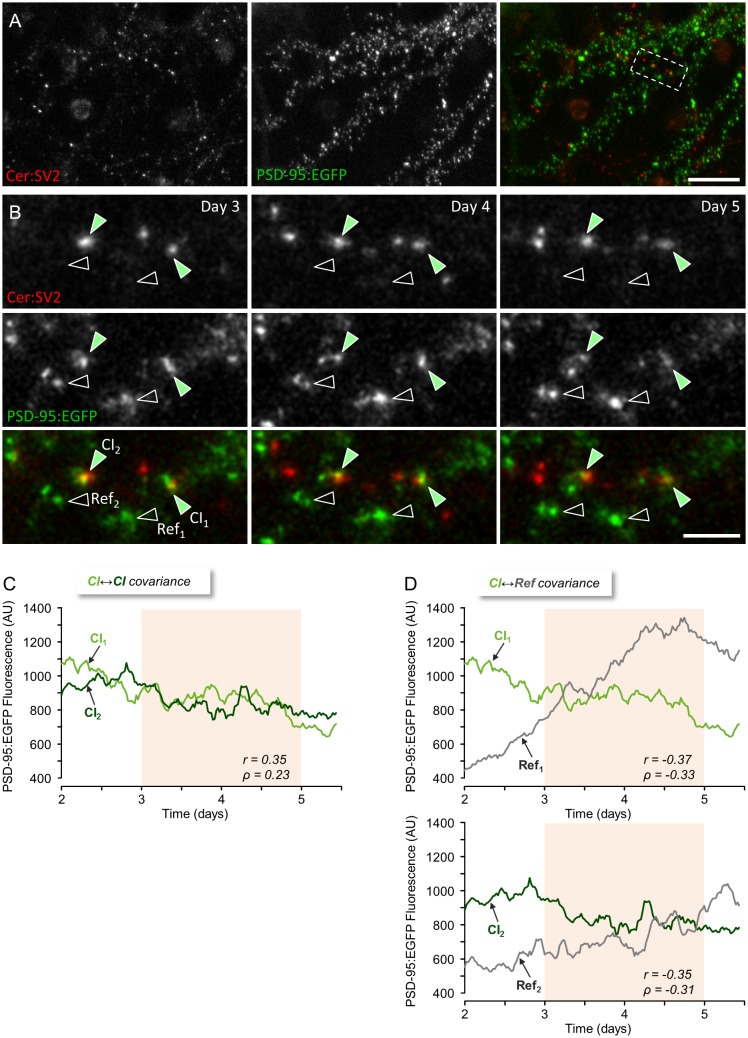
Comparing the size remodeling of CI and non-CI synapses. (A) Cortical neurons growing on an MEA dish expressing cerulean-tagged SV2a (Cer:SV2, a presynaptic vesicle protein) and the postsynaptic scaffolding molecule PSD-95 tagged with EGFP (PSD-95:EGFP). (B) Enlarged view of region enclosed in stippled rectangle in (A) showing two CI synapses (green filled arrowheads) and two Ref synapses (empty arrowheads) at three time points obtained one day apart as part of week-long experiments in which PSD-95:EGFP images were collected at 30-min intervals. (C) Changes in PSD-95:EGFP fluorescence compared for the two CI synapses. (D) Similar comparisons made for each pair of a CI synapse and its respective Ref synapse over the same period. Pearson’s correlation (r) and Spearman’s rank correlation (ρ) coefficients measured for two-day periods (pink background) are shown for each comparison. Bars, 20 μm (A) and 5 μm (B). Source data provided in S1 Data.

We first compared the covariance of CI and non-CI synapses in monolithic cortical networks. In these experiments, individual postsynaptic sites were visualized using PSD-95 tagged with enhanced green fluorescent protein (EGFP) (PSD-95:EGFP; [6,24]), whereas presynaptic sites were visualized using SV2 tagged with Cerulean (a cyan fluorescent protein variant; Cer:SV2; [30]). As shown in Fig 2A, dendrites, individual postsynaptic sites, and presynaptic boutons were readily discernable, allowing us to locate and follow CI and Ref synapses (Fig 2B). To compare the size covariance of CI and non-CI synapses, the networks were mounted on the combined MEA recording/imaging system described above and provided with optimal environmental conditions (a sterile atmospheric environment of 5% CO_2_ and 95% air, slow perfusion with fresh feeding medium, and a temperature of 37°C), allowing us to carry out experiments lasting one week or longer with no signs of deterioration or cell death (Fig 2B). Stacks of images (at 10 focal planes) were collected automatically from 6–12 fields of view (or sites). Images were collected at 30-min intervals for several days concomitantly with recordings of network activity (action potentials) from the 59 electrodes of the MEA dish. As we noted in preliminary experiments that Cerulean exhibited significant photobleaching, axons were imaged at longer intervals (once every 7.5 h). Imaging was started only 2–3 d after mounting the preparations, as we noted here and elsewhere [6,17] that the first 24–36 h of such experiments are invariably associated with increases in spontaneous activity levels related to the introduction of slow perfusion. Imaging in spontaneously active networks was then carried out for at least two further days. Finally, the Na^+^ channel blocker tetrodotoxin (TTX) was added to the MEA and perfusion media to suppress spontaneous network activity, and imaging was continued for additional 1–2 d.

**Fig 3:**
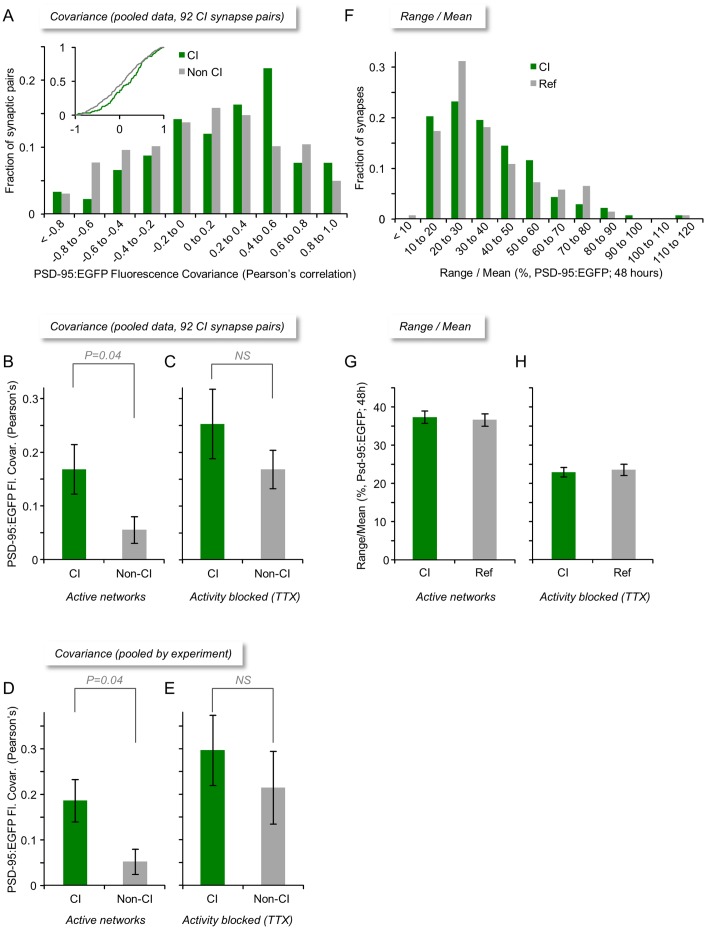
Size remodeling covariance of CI and non-CI synapses in monolithic networks. (A) Distributions of size remodeling covariance values (Pearson’s correlation) for CI and non-CI synapse pairs (92 CI pairs from 24 neurons in 6 experiments). Inset: Same data shown as cumulative histogram. (B,C) Average (±SEM) size remodeling covariance for all CI and non-CI synapse pairs in spontaneously active networks (B) and after suppressing spontaneous activity with TTX (C). (D, E) Same as (B, C)—data pooled by experiment. (F) Distributions of range over mean values for CI and Ref synapses for the same two-day imaging periods. (G, H) Average (±SEM) of range over mean values for all CI and Ref synapses in spontaneously active networks (E) and after suppressing spontaneous activity with TTX (F). Statistical significance values based on two-tailed Mann-Whitney *U* tests. Source data provided in S1 Data.

In agreement with prior cell culture [3,6,11,13,24,31] and in vivo [4,15] studies, the fluorescence of individual PSD-95:EGFP puncta often changed considerably over timescales of many hours. This is exemplified for two CI and two non-CI synapses in Fig 2C. The synapse size covariance of CI and non-CI synapses was then compared by calculating the correlation between the changes in PSD-95:EGFP fluorescence for each CI and non-CI pair over periods of 48 h. This is illustrated for one CI synapse pair (Fig 2C) and respective non-CI pairs (Fig 2D). In this example, the covariance of the CI pair is much greater than that of the non-CI pairs; this difference, however, was not nearly as obvious in all such comparisons (92 pairs from 24 neurons in 6 experiments). In fact, distributions of both Pearson’s correlation coefficients (r) and Spearman’s rank correlation coefficients (ρ) measured for both CI and non-CI pairs were quite broad (Fig 3A and S1A Fig, respectively). Nevertheless, the average covariance measured for all 92 CI pairs was somewhat greater than that measured for all non-CI pairs: (Fig 3B, S1B Fig; CI pairs: r = 0.17 ± 0.05, ρ = 0.15 ± 0.05; non-CI pairs: r = 0.06 ± 0.02, ρ = 0.05 ± 0.02; average ± SEM; *p* = 0.04, *p* = 0.04, Pearson’s and Spearman’s correlation respectively, two-tailed Mann-Whitney *U* test). This difference was also observed when data were pooled by experiment (Fig 3D, S1D Fig; CI pairs: r = 0.19 ± 0.05, ρ = 0.18 ± 0.05; non-CI pairs: r = 0.05 ± 0.03, ρ = 0.04 ± 0.03; average ± SEM; *p* = 0.04, *p* = 0.04, Pearson’s and Spearman’s correlation, respectively, Mann-Whitney *U* test).

If the greater covariance observed for CI synapses is due to the commonality of their activation histories, blocking network activity might be expected to reduce CI synapse covariance to levels observed for non-CI synapses. Somewhat surprisingly, however, suppressing spontaneous network activity as described above, resulted in substantial increases in covariance values for both CI and non-CI synapses (Fig 3C and 3E, S1C and S1E Fig; CI pairs: r = 0.25 ± 0.06, ρ = 0.25 ± 0.06; non-CI pairs: r = 0.17 ± 0.04, ρ = 0.17 ± 0.03; average ± SEM). We attribute this general increase in remodeling covariance to the nonspecific growth of glutamatergic synapses associated with the suppression of network activity (S2A Fig, see also [6,24]). A small difference between the covariance of CI and non-CI pairs was still apparent; this difference, however, was not statistically significant (*p* = 0.33, *p* = 0.41, Pearson’s and Spearman’s correlation, respectively, Mann-Whitney *U* test). The suppression of network activity is known to evoke and affect numerous cellular processes (collectively referred to as synaptic “homeostatic” processes [32]) and parametrically affect the statistics of stochastic remodeling processes [6,16]. As the effects of “homeostatic” processes are not easily disentangled from activity-dependent remodeling processes in active networks, these apparently straightforward experiments were not as informative as might have been expected, although they hint that CI synapses might change in a slightly more correlated manner even when network activity is blocked (see Discussion).

Although the size covariance observed for CI synapses in active networks was somewhat greater than that observed for non-CI synapses, the difference was surprisingly modest. We explored several possible reasons for this modest difference.

We first considered the possibility that the overall extent of remodeling exhibited by synapses in these preparations was small, and, thus, the measures of covariance used here might have reflected, for the most part, the (in)coherence of low amplitude noise in fluorescence measurements. To evaluate this possibility, we measured for each synapse its normalized range of change (“range over mean”) defined as

$$\frac{Range}{Mean}=100*\frac{F_{max}-F_{min}}{\bar{F}}$$

where *F*_*max*_, *F*_*min*_, and $\bar{F}$ are the maximal, minimal, and average PSD-95:EGFP fluorescence intensities, respectively, measured for a given synapse over a period of 48 h. As shown in Fig 3F, distributions of range over mean values were rightward skewed and similar for CI and Ref synapses; about 35% of synapses changed by more than 40% over this period, whereas averages (±SEM) of range over mean values were 37% ± 1.6% (CI) and 37% ± 1.6% (Ref) (Fig 3G). Thus, synapses exhibited substantial changes over these periods, similar in magnitude to changes induced in organotypic slice cultures by paradigms that induce long-term potentiation (33% on average; [18]). This and the fact that all data were low-pass filtered before analysis is thus not in line with the possibility that our covariance measures mainly reflect low amplitude measurement noise (see also [16]). Interestingly, the suppression of network activity reduced, but did not eliminate, synaptic remodeling (Fig 3H; CI: 23% ± 1.2%; Ref: 24% ± 1.5%; average ± SEM). Here too, however, the contributions of “homoeostatic” and other processes to this remodeling are not readily disentangled.

**Fig 4:**
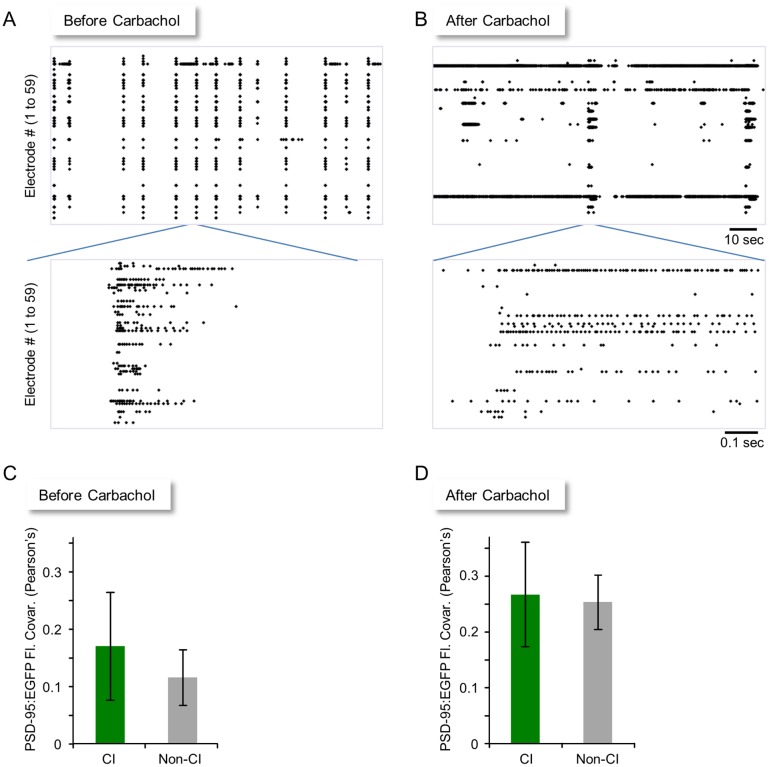
Diversifying network activity by a means of a cholinergic agonist. (A) A 120-s recording of spontaneous activity in a network of cortical neurons growing on a MEA. Each line reports activity (action potentials) recorded from one of the 59 extracellular electrodes of the MEA. Each dot represents one action potential. An enlarged portion of the recording is shown in the bottom panel. Note the tendency of spontaneous activity in these networks to occur as synchronized, network-wide bursts. (B) The same network as in (A) after exposure to 20 μM of Carbachol. (C) Average (±SEM) size remodeling covariance for all CI and non-CI synapse pairs before exposure to Carbachol (27 CI pairs from 8 neurons in 2 experiments). (D) Average (±SEM) size remodeling covariance for all CI and non-CI synapse pairs after exposure to Carbachol. Source data for (C) and (D) provided in S1 Data.

The expected differences in size covariance of CI and non-CI synapses are based on the assumption that activity histories of CI synapses are much more similar than activity histories of non-CI synapses. If, however, all synapses—regardless of their presynaptic origin—share similar activation histories, the size covariance of CI and non-CI synapses might not be expected to differ much. This possibility cannot be ignored, as activity in the preparations used here tends to occur as synchronized bursts that encompass a large fraction of neurons within the network (Fig 4A; e.g., [6,24,33–36]). To increase the “contrast” between the activity histories of synapses belonging to different neurons, we desynchronized network activity by exposing the neurons to Carbachol [24], a non-hydrolysable cholinergic agonist. As shown in Fig 4B, Carbachol (20 μM) greatly diversified the spontaneous activity characteristics, causing some neurons to fire continuously, others to fire more sporadically, and others to fire only occasionally. Furthermore, the tendency of the network to generate network-wide, synchronous bursts was suppressed. Somewhat unexpectedly, this manipulation eliminated the differences between CI and non-CI synapses while elevating their absolute size covariance values (Fig 4C and 4D; CI pairs: r = 0.26 ± 0.09, ρ = 0.21 ± 0.11; non-CI pairs: r = 0.25 ± 0.05, ρ = 0.22 ± 0.06; average ± SEM). Here too, the increased covariance reflects the generalized synaptic growth that follows prolonged exposure to cholinergic agonists (S2B Fig) [24].

The experiments described so far indicated that synapses with similar activity histories changed in a somewhat more correlated manner in comparison to synapses with apparently different activity histories, but the difference between the two groups was rather modest. The possibility that this might have been due to the limited diversity of activity histories in these networks was not supported by pharmacological network desynchronization, but the interpretation of the latter experiments was complicated by the global effects of cholinergic agonists on synaptic properties. Moreover, due to the tendency of synchrony to reemerge after ~12 h in such experiments [24], the duration of such experiments was inherently limited. We thus sought to diversify the activity histories of CI and non-CI synapses by different means. To that end, we turned to modular network architectures.

### Covariance of CI and non-CI Synapses in Modular Networks

**Fig 5:**
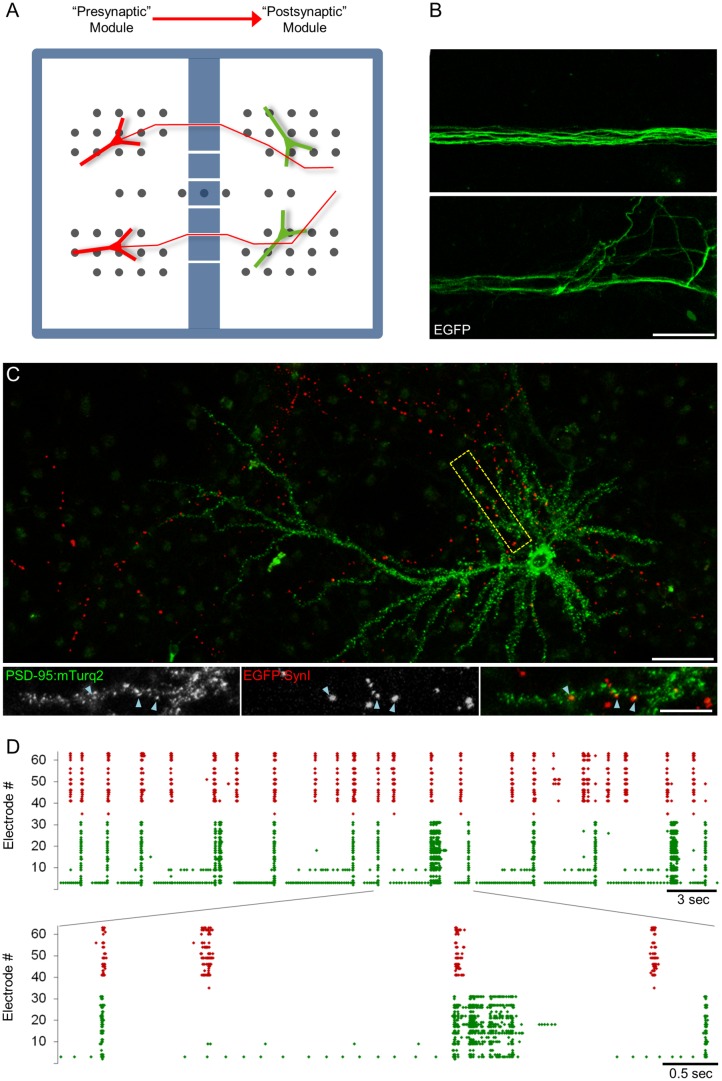
Diversifying network activity by means of modular networks. (A) Schematic diagram of a four-quadrant (4Q) MEA divided into two modules by means of a barrier containing a small number of channels. Neurons in the “presynaptic” module (left) were infected with a lentiviral vector encoding for EGFP:SynI (red). A number of axons crossed over into the “postsynaptic” module in which the expression of PSD-95:mTurq2 was induced in a small number of neurons (green). Note that axons of neurons in the “postsynaptic” module probably also crossed over to the presynaptic module, but these were for the most part invisible. (B) Axons within channels (top) and entering the postsynaptic module (bottom) were visualized by expressing EGFP in the presynaptic module. EGFP expression was done only for development purposes and not used in subsequent experiments. Bar, 20 μm. (C) A neuron in the postsynaptic module expressing PSD-95:mTurq2 (green) innervated by axons originating in the presynaptic module belonging to neurons expressing EGFP:SynI (red). A number of CI synapses within the region enclosed in the stippled rectangle are shown in the bottom panels (cyan arrowheads). Bars, 50 μm (top), 20 μm (bottom). (D) A 40-s recording of activity from the two modules (presynaptic, red; postsynaptic, green). An enlarged (6 s) portion of the recording is shown in the bottom panel. Note that some bursts spread from one module to the other, whereas others did not.

As mentioned above, large groups of neurons in the networks used here tend to fire in synchronized bursts, indicating that the activity histories of neurons in such networks might be quite similar. Previous studies have shown, however, that when such networks are divided into modules separated by barriers partially restrictive to axonal extension, activities in the two modules become more disparate ([37]; see [38] for a comprehensive analysis). We thus set out to compare the size covariance of synapse pairs innervated by axons originating in the same module with the size covariance of synapse pairs in which each synapse is innervated by axons originating in two different modules. To that end, we labeled neurons in one module with a postsynaptic reporter (referred to here as the “postsynaptic” module) and labeled cells in the other module with a presynaptic reporter (the “presynaptic” module; see Fig 5A for a schematic illustration of this “presynaptic/postsynaptic” arrangement). We then searched for pairs of synapses on neurons in the postsynaptic module formed by axons that crossed over from the presynaptic module. The assumption here was that the activity history of these synapses will be similar yet substantially different from the histories of most other synapses in the postsynaptic module, the axons of which were much more likely to have local origins.

In practice, networks were divided into two subnetworks by fabricating polydimethylsiloxane (PDMS) inserts with two compartments and sealing them onto special MEA dishes whose electrodes were arranged in a modular fashion (four-quadrant [4Q] MEAs). The two modules were connected through 6–12 very narrow channels (~400 μm long, ~13 μm wide, and ~3 μm high), which allowed some axons to grow across the barrier and innervate neurons in the other module (Fig 5B), yet were restrictive to the migration of entire cells. Neurons in the presynaptic module were labeled with GFP-tagged Synapsin-Ia (EGFP:SynI) instead of Cer:SV2 due to its much greater photostability, its high endogenous expression levels, and its very high fidelity as a presynaptic marker [39]. Neurons in the postsynaptic module were labeled with PSD-95 tagged with mTurquoise2 (PSD-95:mTurq2), a very bright and relatively photostable variant of cyan fluorescent protein ([40]; see also [13]). The expression of each reporter was fully restricted to its respective module, ensuring that EGFP:SynI-labeled axons observed in the postsynaptic module originated in the presynaptic module. A limited number of EGFP:SynI-labeled axons crossed over to the postsynaptic module (Fig 5B) and formed synapses with neurons in that module (Fig 5C); based on comparisons of axon labeling density in the presynaptic and postsynaptic modules, axons from the presynaptic module represented a tiny fraction (less than 1%) of the total number of axons in the postsynaptic module. Thus, the vast majority of PSD-95:mTurq2 puncta in the postsynaptic module was innervated by axons originating within that module.

The presence of extracellular electrodes in both modules allowed us to examine the disparity of activity in the two modules. As shown in Fig 5D, some network-wide bursts spread from one module to the other (with some delay), but many network-wide bursts remained confined to one module and did not spread to the other module. To verify that axons traversing the barrier indeed carried the activity patterns of the presynaptic module into the postsynaptic module, we expressed the genetically encoded calcium indicator GCaMP6s [41] in presynaptic module neurons and used an electron-multiplying charged couple device (EMCCD) camera to measure Ca^2+^ transients in the presynaptic boutons of axons that crossed into the postsynaptic module (S3 Fig). To that end, sequences of 600 frames were captured at rates of ~7 Hz, allowing us to compare the timing of Ca^2+^ transients with network activities of the presynaptic and postsynaptic modules. As illustrated in S3E Fig, Ca^2+^ transients measured in such axons corresponded extremely well with bursts of activity recorded from the electrodes in the presynaptic module, but not nearly as well with bursts recorded from the postsynaptic module. This analysis also confirmed that Ca^2+^ transients in boutons distributed along the labeled axonal segments correlated almost perfectly, as might be expected. (S3C and S3D Fig; see also [42]). Collectively, these observations show that activity histories of CI synapses are very similar, insofar as action potentials are concerned, whereas those of non-CI synapses differ significantly in both patterning and timing.

We then carried out long-term combined imaging and electrophysiological recordings of neurons expressing PSD-95:mTurq2 and of axons expressing EGFP:SynI as described above. PSD-95:mTurq2 was imaged at 1-h intervals (and EGFP:SynI at 1–3-h intervals) for 2 d. Here too, imaging was initiated only 2–3 d after mounting the preparations on the microscope. After the experiments CI, CI_SD_, and Ref synapses were located, tracked, and their fluorescence values measured. The covariance of CI, CI_SD_, and non-CI pairs (i.e., pairs in which one synapse was innervated by an axon from the presynaptic module and the other by a local axon) was then calculated and compared.

**Fig 6:**
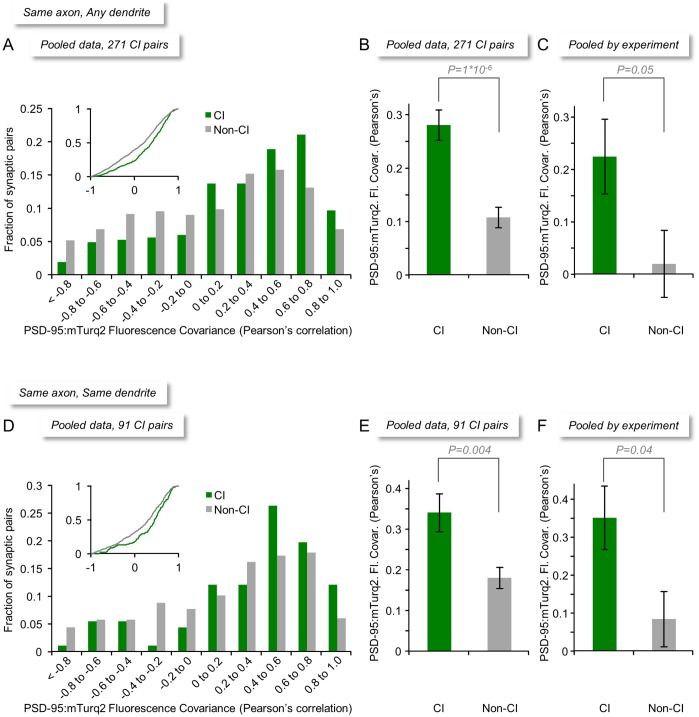
Size remodeling covariance of CI and non-CI synapses in modular networks. (A) Distributions of size remodeling covariance values (Pearson’s correlation) for all CI and non-CI synapse pairs (271 CI pairs from 29 neurons from 8 experiments). Inset: Same data shown as cumulative histogram. (B) Average (±SEM) size remodeling covariance for all CI and non-CI synapse pairs. (C) Same as (B)—data pooled by experiment. (D) Distributions of size remodeling covariance values for all CI_SD_ (that is, same axon, same dendrite) and non-CI synapse pairs (91 CI_SD_ pairs from 29 neurons from 8 experiments). Inset: Same data shown as cumulative histogram. (E) Average (±SEM) size remodeling covariance for all CI_SD_ and non-CI synapse pairs. (F) Same as (E)—data pooled by experiment. Statistical significance values based on two-tailed Mann-Whitney *U* tests. Source data provided in S1 Data.

As in the experiments performed in monolithic networks (Fig 3), distributions of correlation values measured for CI, CI_SD_, and non-CI pairs were quite broad (Fig 6A). Yet, in agreement with the aforementioned experiments, the average covariance measured for all CI pairs was greater than that measured for all non-CI pairs (Fig 6B and S4B Fig; CI pairs: r = 0.28 ± 0.03, ρ = 0.28 ± 0.03; non-CI pairs: r = 0.11 ± 0.02, ρ = 0.11 ± 0.02; average ± SEM; *p* = 1*10^−6^, *p* = 4*10^−7^, Pearson’s and Spearman’s correlation, respectively, Mann-Whitney *U* test; 271 CI pairs from 29 neurons from 8 experiments). This difference was also observed when data were pooled by experiment (Fig 6C, S4C Fig; CI pairs: r = 0.22 ± 0.07, ρ = 0.24 ± 0.07; non-CI pairs: r = 0.02 ± 0.06, ρ = 0.02 ± 0.06, average ± SEM; *p* = 0.05, *p* = 0.04, Pearson’s and Spearman’s correlation, respectively, two-tailed Mann-Whitney *U* test). A similar observation was made for CI_SD_ pairs, i.e., nearby synapses innervated by the same axon and formed on the same dendrite (Fig 6D and 6E, S4D and S4E Fig; CI pairs: r = 0.34 ± 0.05, ρ = 0.34 ± 0.05; non-CI pairs: r = 0.18 ± 0.03, ρ = 0.16 ± 0.03; average ± SEM; *p* = 0.0036, *p* = 0.0008, Pearson’s and Spearman’s correlation, respectively, two-tailed Mann-Whitney *U* test; 91 CI_SD_ pairs from 29 neurons from 8 experiments). This difference was also observed when data were pooled by experiment (Fig 6F, S4F Fig; CI pairs: r = 0.35 ± 0.08, ρ = 0.36 ± 0.08; non-CI pairs: r = 0.08 ± 0.07, ρ = 0.06 ± 0.07, average ± SEM; *p* = 0.04, *p* = 0.04, Pearson’s and Spearman’s correlation, respectively, two-tailed Mann-Whitney *U* test).

Although the introduction of a barrier diversified the activity histories of synapses belonging to non-CI pairs, some network-wide bursts did spread from one module to the other, suggesting that activity histories of synapses belonging to non-CI pairs were not entirely dissimilar. The degree to which the two modules were coupled in terms of their bursting activity varied from one experiment to another, ranging from 0.20 to 0.91 (0.64 ± 0.26 average ± standard deviation; see Materials and Methods for further details on this measure). Comparing this coupling with non-CI synapse covariance on an experiment-by-experiment basis revealed a positive correlation (r = 0.62) between these two measures, although this correlation was not statistically significant (*p* = 0.09). In contrast, and as might be expected, no correlation was observed for CI synapses (r = 0.04, *p* = 0.99). It should be noted that the measure used here to quantify coupling only considered the fraction of bursts that propagated from one module to another, ignoring functionally important features such as propagation delays and burst durations (see [38] for a comprehensive analysis). Nevertheless, these findings indicate that even in modular networks, the size covariance of non-CI synapses might be influenced somewhat by partial similarities in activity histories, although this influence is at most very small (Fig 6B, 6C, 6E and 6F, S4B, S4C, S4E and S4F Fig; see Discussion).

Although the remodeling covariance of CI and non-CI pairs differed in a statistically significant manner, the actual differences were rather modest. We wondered if this might be due to the inclusion of relatively small synapses, which are more prevalent than large synapses in these preparations [6,16] and in the intact brain [43], as these would be most sensitive to minor fluctuations in background fluorescence or measurement noise. To examine this possibility, we increased the stringency of selection criteria of CI pairs, removing small synapses from the analyses. Even with these stringent selection conditions, however, differences between the covariance of CI and non-CI pairs remained quite modest (S5 and S6 Figs; 103 CI pairs: r = 0.26 ± 0.05, ρ = 0.26 ± 0.04 non-CI pairs: r = 0.08 ± 0.03, ρ = 0.08 ± 0.03; *p* = 0.003, *p* = 0.002, Pearson’s and Spearman’s correlation, respectively; 40 CI_SD_ pairs: r = 0.33 ± 0.07, ρ = 0.35 ± 0.06; non-CI pairs: r = 0.17 ± 0.04, ρ = 0.14 ± 0.04; *p* = 0.04, *p* = 0.009, Pearson’s and Spearman’s correlation, respectively; Mann-Whitney *U* test).

**Fig 7:**
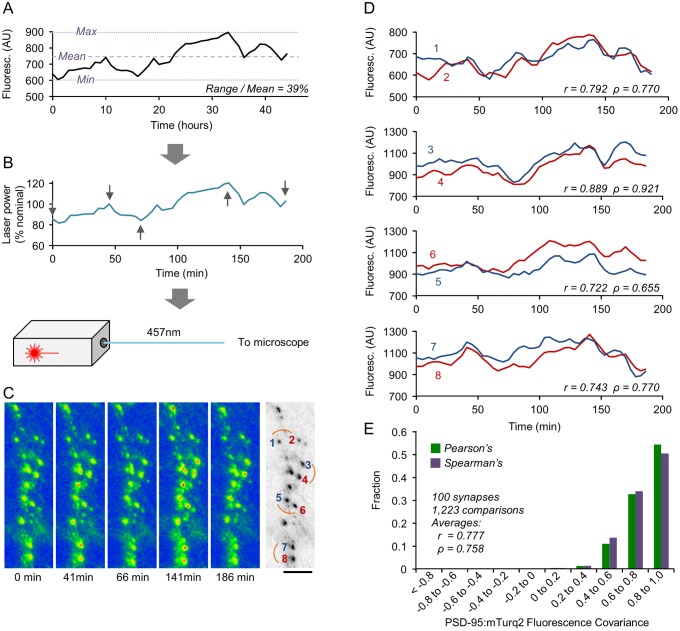
Measuring maximal detectable covariance values. (A) A synapse selected to serve as a template for excitation light intensity modulation, based on a range over mean value similar to the average range over mean values measured over 48-h periods for PSD-95:EGFP and PSD-95:mTurq2 puncta belonging to CI synapses; (~37%; Fig 3G and ~35%, not shown, respectively). (B) The intensity of the excitation laser was modulated to match changes in the fluorescence of selected synapses such as that shown in (A). Intensity was modulated around nominal laser intensities (expressed here as 100%). (C) Pseudocolor images of a segment of a dendrite expressing PSD-95:mTurq2 at five time points (gray arrows in B). The image on the right is an inverted grayscale of the first image (t = 0). Bar, 10 μm. (D) Comparisons of fluorescence covariance for pairs of PSD-95:mTurq2 puncta connected by short arcs in (C) (right-hand side). Note the marked but imperfect fluorescence covariance for such pairs. (E) Distribution of covariance values (Pearson’s and Spearman’s correlations) for 1,223 comparisons made for 100 synapses from 4 different neurons. Source data for (E) provided in S1 Data.

Not only were the differences in size covariance for CI and non-CI synapses rather modest; the absolute covariance values for CI synapses were surprisingly small, with the highest average values observed in any of the experiments described above being r = 0.35 and ρ = 0.36 (CI_SD_ synapses in modular networks; Fig 6F, S4F Fig, respectively). This would seem to suggest that, in addition to joint remodeling, each synapse within a CI pair exhibits significant change that occurs independently of its counterpart. Assuming that CI synapses and, in particular, CI_SD_ synapses share common activity histories, the residual remodeling would seem to represent spontaneous, activity-independent synaptic remodeling. Yet it remained possible that at least some of the imperfect size covariance of CI synapses stems from measurement limitations, such as fluorescence measurement inaccuracies. We therefore set out to determine what would have been the average size covariance measured in our system had CI synapse sizes co-varied perfectly. To that end, we introduced artificial correlations between PSD-95:mTurq2 puncta synaptic fluorescence levels by modulating excitation light intensities from one time point to the next (Fig 7A and 7B); we then measured the fluorescence of PSD-95:mTurq2 puncta (Fig 7C) and calculated the correlations for all pairs of synapses in the fields of view (Fig 7D). The depths and temporal profiles of excitation laser light intensity modulation were based on changes in fluorescence levels measured for particular synapses during the long-term experiments described above (Fig 7A), selecting for this purpose synapses whose range/mean ratios were similar to average range/mean ratios measured during those experiments (e.g., Fig 3F and 3G). The experiments were carried out in exactly the same way all experiments described so far were performed, except that here, 48 images were collected in rapid succession to minimize the effects of true synaptic remodeling. As shown in Fig 7E, average correlation values measured here were all positive and rather high (r = 0.78, ρ = 0.76; 100 synapses from 4 neurons, 1,223 pairwise comparisons). These experiments thus suggest that the modest size covariance observed for CI synapses cannot be solely attributed to measurement inaccuracies.

### Size Similarity of CI Synapses

**Fig 8:**
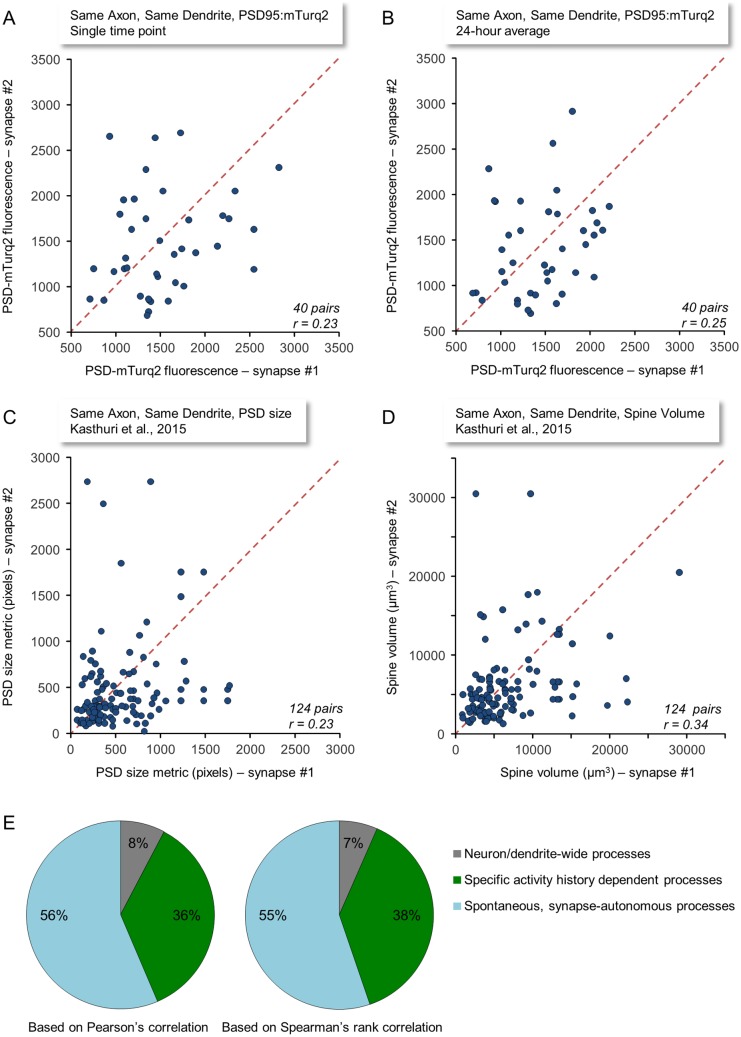
Size similarity of synapses belonging to the same CI_SD_ pairs. (A) PSD-95:mTurq2 fluorescence values for synapses belonging to the same CI_SD_ pairs. Each point represents the PSD-95:mTurq2 fluorescence at the first time-point of a time lapse series. Data shown are for the most stringently selected CI_SD_ pairs (40 pairs; see also S5 and S6 Figs). (B) Same as (A) except that here data points represent fluorescence values averaged over 24-h time periods (24 measurements). (C) A similar analysis performed for PSD sizes of 124 pairs of CI_SD_ synapses taken from the data set published by [21]. Each point represents a comparison of the PSD sizes (in originally published metrics) of one such pair. To locate such pairs, the data set containing detailed information on 1,700 synapses was filtered to locate synapses connecting the same axons and the same dendrites. Shaft synapses or synapses for which no spine volume data were provided were excluded. Eighty-five CI_SD_ synapse groups composed of pairs (72), triplets (10), quadruplets (2) and quintuplets (1) were found. Groups containing more than two synapses were broken into all possible pairwise comparisons. (D) Same as (C) but for spine volumes (in μm^3^). (E) Contributions of specific activity histories, neuron/dendrite-wide processes, and spontaneous processes to glutamatergic synapse remodeling. The fractional contributions were calculated as explained in main text. Source data for (A–D) provided in S1 Data.

So far, the analyses presented concerned the degree to which sizes of synapses with common activity histories changed together over time. But how similar were the absolute sizes of such synapses? It might be expected that, given their common activity history, their sizes should be similar [21,22]. To examine the degree to which sizes of synapses with identical activity histories were similar, we plotted for each synapse in a CI_SD_ synapse pair its PSD-95:mTurq2 fluorescence against the fluorescence of its counterpart in the same pair. For this analysis we used the most stringent data set in which the smallest synapses were omitted (see S5 and S6 Figs), using the measures of PSD-95:mTurq2 fluorescence obtained from the first image stack of each time-lapse series. As shown in Fig 8A, the correlation between the sizes of synapses belonging to the same CI_SD_ pair was rather poor (r = 0.23). We then repeated the same analysis for the same synapses, but now using PSD-95:mTurq2 fluorescence values averaged for each synapse over a period of 24 h. Here too, however, the correlation was still quite poor (Fig 8B; r = 0.25).

The degree to which synapses formed between the same axon onto the same dendrite have similar sizes was explored as part of a recent study in which a small volume of mouse neocortex was reconstructed in full by serial section electron microscopy [21]. All data obtained in that study were made publicly accessible, allowing us to compare our findings, obtained in living cells, in cell culture, by light microscopy, to data obtained in fixed tissue, in vivo, by means of state-of-the-art electron microscopy. To that end, we identified in the aforementioned data set groups of spine synapses made by a particular axon onto a particular dendrite (CI_SD_ synapses) and plotted, for each synapse in each CI_SD_ pair, its PSD size and spine volume against the PSD size and spine volume of its CI_SD_ counterpart (124 pairs; Fig 8C and 8D, respectively). As these figures show, the size similarity of in vivo CI_SD_ synapses was no greater than the similarity of CI_SD_ synapses in culture. In fact, the correlation (r = 0.23) between PSD sizes for synapses belonging to the same CI_SD_ pair was identical to the correlation observed in our study for PSD-95:mTurq2 fluorescence at synapses belonging to the same CI_SD_ pair (Fig 8A and 8B).

### Contributions of Specific Activity Histories and Spontaneous Processes to Glutamatergic Synapse Remodeling

The data presented here suggest that the covariance of size changes for synapses that share similar activity histories is greater than that of synapses formed on the same neurons or dendrites that differ in their activity histories. At the same time, the data suggest that the covariance of size changes for synapses that share similar activity histories is significantly smaller than what might have been expected if synaptic remodeling was solely determined by activity histories. Our data thus allow for a conservative estimation of the maximal relative contribution of activity history-dependent processes to glutamatergic synapse remodeling in our system (Fig 8E). For this estimation, we used (1) the highest average covariance values obtained here for CI_SD_ synapses (pooled data, r = 0.34 and ρ = 0.34; Fig 6E and S4E Fig, respectively), as these represent synapses whose activity histories are probably the most similar in our data sets; (2) the lowest average covariance values obtained here for non-CI synapses (pooled data, r = 0.06 and ρ = 0.05; Fig 3B and S1B Fig, respectively), as these represent the lowest possible contributions of (postsynaptic) neuron/dendrite-wide, nonspecific processes (and possibly of some residual shared activity); and (3) the maximal average correlation measurable in our system (r = 0.78, ρ = 0.76; Fig 7E). Using Pearson’s and Spearman’s correlations, respectively, the relative contributions of specific activity histories might thus be estimated as follows:

$$\frac{0.34-0.06}{0.78}\approx 0.36\;\left(\text{Pearson}’\text{s}\right),\text{ and }\frac{0.34-0.05}{0.76}\approx 0.38\;\left(\text{Spearman}’\text{s}\right)$$

The contributions of spontaneous processes that occur autonomously at each synapse can then be estimated as follows:

$$\frac{0.78-0.34}{0.78}\approx 0.56\;\left(\text{Pearson}’\text{s}\right),\text{ and }\frac{0.76-0.34}{0.76}\approx 0.55\;\left(\text{Spearman}’\text{s}\right)$$

This analysis suggests that under our experimental conditions, the ratio of contributions by activity history-dependent and -independent processes to synaptic remodeling is, at most, about 2:3. Put differently, the “signal-to-noise ratio” of activity history-dependent synapse remodeling is approximately 0.7, i.e., less than one.

## Discussion

In the current study, we set out to compare the contributions of specific activity histories to the size remodeling of glutamatergic synapses, with the contributions of processes occurring irrespective of specific histories. To that end, we examined the covariance of changes in the sizes of CI synapses, that is, synapses formed between the same axon and the same neuron or dendrite, under the assumption that the specific activity histories of such synapses will be very similar; we then compared this covariance to that of synapses formed between different axons (non-CI synapses), which presumably differ in their activity histories, and to the maximal covariance measurable in our experimental system. We found that the size covariance of CI synapses was higher than that of non-CI synapses in both monolithic and modular networks; yet the average covariance of CI synapses was rather modest in comparison to what might have been expected had remodeling been dictated exclusively by specific activity histories. Indeed, comparisons of the momentary and time-averaged sizes of CI synapses revealed that the sizes of synapses with nearly identical activity histories correlated rather poorly, in perfect agreement with electron microscopy-based measurements of CI synapse sizes in the intact mouse cortex. A conservative compilation of covariance data for CI and non-CI synapses, and comparisons with maximal covariance values measurable in our system, suggests that only about 40% of glutamatergic synapse size remodeling could have been attributed to specific activity histories, and thus the contributions of other processes, including neuron-wide, nonspecific processes and other, possibly stochastic, synapse-autonomous processes, were at least as great.

### Methodological Considerations

The interpretation of the experiments described here is based on several key assumptions that warrant some discussion.

The first assumption concerns the identification of CI synapses as such. This identification was based on the juxtaposition of pre- and postsynaptic synaptic proteins tagged with fluorescent groups and, thus, ultimately on light microscopy. Several studies (e.g., [21,44]) have suggested, however, that the proximity of an axon to a dendrite or a spine observed by light microscopy might not reliably predict the presence of a synapse, not even in a statistical sense (e.g., [45]). Yet it should be noted that this conclusion pertained to proximities of axons and spines, visualized by volume-filling dyes or electron microscopy, whereas, here, the presence of a synapse was deduced from the juxtaposition of fluorescent foci, originating in proteins that cluster almost exclusively at pre- and postsynaptic sites; thus, the presence of a synapse was deduced here not only from physical proximity but also from the juxtaposition (in three dimensions) of pre- and postsynaptic specializations. Furthermore, unlike most proximity-based assignments, which are typically performed in fixed tissue, assignments here were based on multiple observations of the same clusters over at least 2 d, and thus small movements of axons and dendrites (which are quite common in these preparations; see [46,47]) allowed us to exclude juxtaposed pre- and postsynaptic protein clusters that did not move in unison. It is also worth noting that when we restricted the analysis to the most cleanly identifiable, bright CI synapses, the results were practically identical (compare S5 and S6 Figs with Fig 6, S4 Fig). Thus, although we cannot exclude the possibility that some CI synapses were not really innervated by the same axon, it is unlikely that our conclusions were significantly affected by erroneous assignments.

The second assumption concerns the relationships between tagged PSD-95 fluorescence, synapse size, and synaptic strength. A good correspondence between spine volume and synaptic strength has been established in multiple studies (e.g., [48–51]). Similarly, good correspondences between spine volume, PSD size, as well as α-amino-3-hydroxy-5-methyl-4-isoxazolepropionic acid (AMPA)-type glutamate receptor content have been shown repeatedly (e.g., [18,22,52–54]; reviewed in [23,55]). Finally, an excellent correspondence between tagged PSD-95 fluorescence, measured by light microscopy, and PSD area, measured for the same synapses by electron microscopy, was recently reported [15]. As PSD area is thought to correlate with the number of synaptic glutamate receptors, [56] tagged PSD-95 fluorescence might represent an acceptable surrogate of synaptic strength [55]. Yet, under some circumstances, for example after strong stimuli that drive spine enlargement, spine volume is temporarily decoupled from PSD size and postsynaptic scaffold molecule contents, which “catch up” on a slower timescale (2–3 h; [18,19]); this uncoupling might indicate that synaptic strength is not always predicted correctly by PSD size; by extension, it remains possible that synaptic strength is more stable than measurements of PSD-95 content would seem to suggest, because other processes (for example, changes in glutamate receptor numbers) acting over faster timescales maintain synaptic function within precise limits. How such processes might achieve this over a background of varying scaffold size, however, is not clear. Furthermore, repeated electrophysiological measurements of the same synaptic connections point to significant spontaneous fluctuations in connection strengths over comparable timescales (12 h or less), even when activity or synaptic transmission is blocked pharmacologically (e.g., [57–59]). It thus seems more likely that tagged PSD-95 fluorescence measurements such as those used here and elsewhere provide low-pass filtered estimates of synaptic strength, which underestimate, rather than overestimate, fluctuations in synaptic strength. In this respect, it is worth noting that synaptic contents of AMPA-type glutamate receptors seem to fluctuate at least as much as synaptic PSD-95 contents do ([11]; see also [60]).

The third assumption concerns the premise that CI synapses have similar activation histories when these are integrated over many days. Although this is a very reasonable premise [21,22], it is not perfect. Ignoring for the moment the statistical nature of neurotransmitter release (which would probably average out over these long timescales; [22]), presynaptic sites of cultured hippocampal neurons have been shown to exhibit significant functional variability even for sites located along the same axons (e.g., [61–65]; reviewed in [20]). As a result, activity histories of CI synapses might not be as similar as presumed, which might partially explain the modest covariance of their remodeling. We note, however, that this is an unlikely explanation. First, it was shown that presynaptic functional properties of nearby synapses formed by the same axons on the same dendrites are much more similar than those formed on different dendrites [61,63]. Consequently, had presynaptic variability been at the source of differential remodeling, the covariance of CI_SD_ synapse remodeling should have been much higher than that observed for CI synapses. Such a difference, however, was not apparent (compare Fig 6B, 6C, 6E and 6F; S4B, S4C, S4E and S4F Fig). Along this line, measurements made in cortical neurons suggested that release properties of presynaptic sites formed by the same axons on the same neurons are remarkably similar, even when such synapses are formed on different dendrites of target neurons (a phenomenon referred to as Normalization of Release Probability; [42]). Perhaps more importantly, however, here (Fig 3) and elsewhere (e.g., [5,6,11,13,66]), it was shown that synaptic remodeling continues at significant rates even when activity and/or synaptic transmission is blocked. It therefore seems more likely that the modest remodeling covariance observed for synapses belonging to the same CI pairs is due to spontaneous remodeling processes occurring autonomously at each synapse, independent of activity, specific or otherwise. In fact, we suspect that the variability of presynaptic functional parameters observed in studies such as those mentioned above might be the outcome, rather than the cause, of such spontaneous remodeling processes. This would not be surprising, given the strong coupling between PSD and active zone remodeling (e.g., [13,18,67,68]; see also [52,59,69]). Moreover, the skewed (heavy tailed) distributions of presynaptic properties [61–65,69] as well as previously reported features of active zone remodeling dynamics [13,16] are archetypical hallmarks of a stochastic process known as the Kesten process, which was previously shown to capture the spontaneous remodeling processes of excitatory [16] and inhibitory [17] synapses.

The fourth concerns the assumption that the differences between CI and non-CI synapse covariance stemmed entirely from differences in commonality of activation histories. Even in modular networks, however, some network-wide bursts did spread from one module to the other, suggesting (as elaborated on above) that activity histories of non-CI synapses were not entirely dissimilar. This might have led us to overestimate the covariance contributed by (postsynaptic) neuron/dendrite-wide, nonspecific processes (non-CI synapse remodeling; gray regions in Fig 8E). We note, however, that the very low values of these estimates (7% to 8%) leaves little room for lowering them further. Conversely, the experiments described in Fig 3 and S1 Fig hint that, even in inactive networks, remodeling covariance of CI synapses might still slightly exceed that of non-CI synapses. It thus remains possible that some of the covariance exhibited by CI synapses reflects contributions of factors other than common activity histories. Thus, for example, spontaneous neurotransmitter release from presynaptic boutons belonging to the same axons could be coordinated by a variety of intra-axonal processes, such as molecular and synaptic vesicle interchange [2,70]. Similarly, processes acting extracellularly (such as receptor spillover and retrograde messengers) might act preferably on CI synapses even in the absence of overt activity. Consequently, we may have overestimated the contributions of specific activity history-dependent processes to synaptic remodeling (Fig 8E, green sectors). It is important to note, however, that neither of these deviations from the underlying assumptions would affect our estimations of the largest component, that is, synapse-autonomous, spontaneous remodeling processes (Fig 8E, blue sectors), and, thus, these deviations were unlikely to significantly impact the main conclusions of this study.

Finally, it should be noted that our experiments were carried out in networks of dissociated rat cortical neurons in primary culture. In the context of this study, the system was advantageous not only because of the experimental access it provided but also because it allowed us to focus on activity history dependence of synaptic remodeling in a manner free from other influences such as neuromodulation. Yet, it might be asked to what degree the conclusions reached here apply beyond our experimental system. As described in the introduction, the observation that spine volumes and PSD sizes fluctuate in the intact brain is well documented. Where such observations are concerned, however, it remains unknown what fraction of these fluctuations represents bona fide history-dependent synaptic remodeling and which represents other, possibly stochastic, processes. Nevertheless, we note that history-dependent remodeling processes should ultimately control synaptic size, and when sizes of synapses with apparently identical histories are compared, their sizes correlate quite poorly, not only in our data but in the intact mouse neocortex as well (Fig 8; [21]). A similar finding emerges from another recent electron microscopy study for a much smaller data set (17 pairs of CI synapses from adult rat hippocampi [22]). Here, it was found that, on average, within each CI pair, spine volumes and PSD areas differed by factors of ~2 and ~3, respectively. We thus cautiously suggest that our findings, obtained in culture, might apply to the intact brain as well, although the actual fraction of activity history-dependent remodeling might differ somewhat and vary, perhaps, according to behavioral state.

### Fractional Contributions of Activity History-Dependent and -Independent Processes

The assertion that synaptic strength is defined by the history of pre- and postsynaptic activity is one of the oldest, yet widely accepted tenets of contemporary neuroscience [71,72]. One facet of this assertion concerns activity-dependent structural plasticity of synaptic connections, including changes in the sizes of existing synapses [73]. Indeed, the capacity of particular activity paradigms to drive excitatory synapse enlargement (and shrinkage) is now well established (reviewed in [23]). The stimulation paradigms used in such studies, however, are typically brief and artificial (e.g., tetanic or theta burst stimulation, glutamate uncaging in low extracellular Mg^2+^, sometimes in the presence of various pharmacological agonists). In contrast, relationships between histories of protracted, more natural activity forms and synapse remodeling are less established. In the current study, we show that sizes of synapses with shared activity histories co-vary more than sizes of synapses with different activity histories (Figs 3 and 6, S1 and S4 Figs), thus demonstrating that histories of spontaneously occurring activity forms can significantly affect synaptic remodeling as well.

Somewhat surprisingly, our data also suggest that the contributions of shared activity histories and the contributions of spontaneous processes to synaptic size remodeling are of comparable magnitudes. It might be argued that other activity regimes, which differ from those present in the networks studied here, might be more effective in controlling synaptic sizes. Had this been the case, however, it might have been expected that sizes of CI synapses would be more similar in vivo as compared to the situation in culture, given that activity regimes in vivo are richer and more physiologically relevant. Contrary to these expectations, however, sizes of CI synapses in vivo were no more similar than the sizes of CI synapses in culture (r = 0.23; Fig 8). It might be further argued that this poor correlation is attributable to local details such as bouton to bouton variability, as described above. We note, however, that such sensitivity to local details would further undermine the notion that predictable relationships exist between synaptic remodeling and particular histories of pre- and postsynaptic activities. We thus suspect that, regardless of activity regime, the governance of synapse remodeling by particular activity histories is partial at best. In this respect, it is worth noting that the magnitude of PSD enlargement induced by the aforementioned experimental stimuli (50% or less; e.g., [18,19,74]) is not that different from the magnitude of spontaneously occurring changes in PSD sizes observed in the intact cortex of mice merely maintained in their home cages (44% on average; [15]). It is also worth noting that both covariance measures used here (i.e., Pearson’s correlation and, even more so, Spearman’s rank correlation) only quantify similarities in the trends of synaptic remodeling but are quite indifferent to the similarities in the absolute magnitudes of such remodeling. Thus, the governance of synapse remodeling by particular activity histories might be even more limited than our estimates indicate.

How then can one reconcile the overwhelming evidence for activity history-dependent synapse remodeling with such a significant degree of spontaneous remodeling? How can persistent functions be embedded in neuronal networks if directed and spontaneous changes in synaptic sizes are of similar magnitude? This experimental and conceptual gap might be partially bridged by considering the following matters.

The first matter concerns the growing appreciation that synaptic plasticity is affected or even gated by various neuromodulatory systems [72,75,76]. The absence of neuromodulatory systems in the networks used here was useful to examine the net contributions of particular activity histories; yet, in the intact brain, timed neuromodulator release might significantly enhance the contributions of specific activity histories and thus minimize the relative contributions of spontaneous remodeling, at least during behaviorally important time windows. We note once again, however, that this entails an expectation that CI synapse sizes would be quite similar in vivo, an expectation that is not matched.

A second matter concerns the fact that functional connections between neurons are often based on multiple synapses (reviewed in [20]), and, thus, activity history-independent fluctuations at individual synapses might average out at the level of neuron-to-neuron connections. Furthermore, changes in connection strength might be most reliably modified by increasing or decreasing the number of synapses connecting two neurons (e.g. [21]; see also [77]). Indeed, it has recently been shown that numbers of synapses formed between particular axons and dendrites are very different from what might be expected by chance [21,44]. It remains to be seen, however, if the time course over which synapses are added/removed, the actual numbers of such synapses, and the signal-to-noise ratios of multiple synapse connections can satisfactorily address the discordance described above. In this regard, it worth noting a recent in vivo (mouse) study in which basal rates of spine formation and loss were found to be almost unaffected by chronic blockade of calcium channels and N-methyl-D-aspartate (NMDA) receptors [66].

A third matter to consider is the possibility that persistent changes in network function involve vast numbers of synapses and neurons such that fluctuations at the individual synapse level are mitigated by massive redundancy [78] or rendered insignificant by the sheer numbers of synapses involved. Indeed, a recent study provided evidence suggesting that the acquisition of a new motor skill in mice involves about 4,700 motor cortex neurons and about 410,000 synapses [79]. In this regard, it is interesting to note that in his influential monograph, Hebb [73] considered this matter and suggested that, although stochastic processes might preclude predictable actions in small parts of the system, statistical constancies might emerge in larger systems. Indeed, when large numbers of synapses are followed over time, their remodeling dynamics do seem to obey certain well-defined statistical rules [5,8,16].

A final matter to consider is the possibility that stochastic changes in synaptic properties are crucially important components in the organization of network learning, as they enable networks to explore and sample synaptic configurations for those most congruent with input from the external world or with desired functions [80]. This recent study suggests that changes in synaptic weights are driven not only by deterministic, activity-dependent rules (and biological constraints) but also by stochastic processes, which dramatically improve the ability of networks to generalize and compensate for unforeseen changes. Within this context, our finding that the magnitudes of deterministic and stochastic components are comparable would seem to suggest that the contribution of exploratory processes is at least as significant as the contribution of deterministic processes, lending further support to this emerging view of synaptic plasticity.

## Materials and Methods

### Ethics

All experiments were performed in primary cultures of rat neurons prepared according to a protocol approved by the "Technion, Israel Institute of Technology Committee for the Supervision of Animal Experiments" (ethics approval number IL-019-01-13).

### Monolithic MEAs

The thin glass MEAs (MultiChannelSystems—MCS, Germany) used here for monolithic networks contain 59 flat, round electrodes made of titanium nitride arranged in an 8 x 8 array with an inter-electrode spacing of 200 μm. In this arrangement, the corner electrodes are missing, and one of the leads is connected to a large reference (ground) electrode. Although the recording and reference electrodes are opaque, the very thin glass (180 μm) substrate and the Indium Tin Oxide leads are fully transparent, allowing excellent optical access to the cells growing on the array.

### Fabrication of Modular MEAs

Modular MEAs were prepared using 4Q, commercially available MEAs (MultiChannelSystems) fabricated to our request on thin glass. Apart from their layout, 4Q MEAs used here were identical to the thin glass MEAs described above. A PDMS insert was sealed onto the MEA surface, effectively dividing the MEA into two modules, separated by a number of thin channels (similar to the method described in [38]). The PDMS inserts were made using a silicon mold microfabricated using standard, single-layered SU8 photolithography techniques [81,82]. Briefly, SU-8 2002 (Microchem, Inc.) was spun on a 4-inch silicon wafer at a nominal thickness of 3 μm, baked, exposed with a dark-field transparency channel mask, baked again, and developed. Each mold had multiple barrier patterns with channel numbers ranging from 6 to 12 (3 μm x 13 μm x 400 μm; H x W x L). The mold was silanized ([tridecafluoro-1,1,2,2-tetrahydroocytl]-1-trichlorosilane evaporated for 1h in vacuum) to allow easier release and slowly filled with PDMS silicone rubber (Sylgard 184; 10:1 ratio of pre-polymer [base]: cross-linker [curing agent]; Dow-Corning, Midland, Michigan), to 2-mm height and de-gassed in a vacuum desiccator. Once the PDMS spread over the entire wafer, it was cured for 3h at 65°C. Following curing, 17-mm diameter circular barriers were cut, and two 5-mm-wide wells were punched on each side of the channels; the finalized inserts were then stored for future use. On the day of cell culture preparation, each barrier was aligned to the electrodes of pre-coated 4Q MEA dishes (see below) using a drop of 70% ethanol and heated for 2h at 54°C to allow ethanol evaporation and PDMS sealing. Finally, the dishes were cooled to 37°C in a cell culture incubator.

### Neuronal Cell Cultures

Primary cultures of rat cortical neurons were prepared as described previously [6]. Briefly, cortices of 1–2-d-old Wistar rats of either sex were dissected and dissociated by trypsin treatment followed by trituration using a siliconized Pasteur pipette. For monolithic cultures, a total of 1–1.5 x 10^6^ cells were plated onto thin-glass MEA dishes, the surfaces of which had been pretreated with polyethylenimine (PEI, Sigma) to facilitate cell adherence. Modular cultures were prepared on 4Q thin glass MEAs described above (see also [37]) as follows: 100 μl aliquots of cells in suspension (at 1–1.5 x 10^6^/ml cells) were infected with predetermined amounts of viruses and incubated for 2 h in a tissue culture incubator at 37°C. Following the incubation, the infected cells were spun down for 60 s at 2,000 g, and 60 μl of the supernatant were replaced with pre-warmed culture medium, and the cells were resuspended by gentle pipetteation. The process was repeated two more times (three washes in total). After the third spin down and resuspension, the cells were pipetted thoroughly, and 20–25 μl of cells in suspension were seeded in their respective module. No contact was allowed between the two droplets. 160 μl of uninfected cells at similar concentrations were seeded dropwise at the dish perimeter (outside the PDMS barrier) to enrich the environment with diffusive nutritional factors. Dishes with droplets were put in 10-cm petri dishes containing small vessels with water (to maximize humidity) and incubated overnight in a humidified tissue culture incubator at 37°C in a gas mixture of 5% CO_2_, 95% air. The next morning, 2 ml of culture medium were added to each dish.

Both uniform and modular preparations were kept in a humidified tissue culture incubator and grown in medium containing minimal essential medium (MEM, Sigma), 25 mg/l insulin (Sigma), 20 mM glucose (Sigma), 2 mM L-glutamine (Sigma), 5 mg/ml gentamycin sulfate (Sigma), and 10% NuSerum (Becton Dickinson Labware). Half of the volume was replaced three times a week with feeding medium similar to the medium described above but devoid of NuSerum, containing a lower L-glutamine concentration (0.5 mM) and 2% B27 supplement (Invitrogen).

### DNA Constructs, Lentivirus Production, and Transduction

All DNA constructs (except GCaMP6s; see below) were introduced into neurons using third generation lentiviral expression vectors based on the FUGW backbone [83]. The construct used for expressing PSD-95:EGFP (FU-PSD-95:EGFP-W) was described in detail in [6]. The construct used to express Cer:SV2 (FU-Cer:SV2a-Wm) was made as follows: FUGW was modified to FUGWm by moving the XhoI site from the 3’ to the 5’ side of the woodchuck hepatitis post-transcriptional regulatory element (WPRE). Cerulean [30], flanked with AgeI (5') and BsrGI (3') sites, was synthesized de novo and inserted into FUGWm instead of EGFP using the AgeI and BsrGI sites, resulting in the interim construct FUCWm. SV2a was then cut out of FU-EGFP:SV2a (a generous gift by Craig C. Garner; [84]) using BsrGI (5’) and XhoI (3’) sites and inserted into FUCWm, resulting in FU-Cer:SV2a-Wm. Sequencing confirmed 100% identity with Rattus norvegicus SV2A (GenBank accession: L01788.1). The construct used to express PSD-95:mTurq2 was made as follows: Large-scale gene synthesis was used to synthesize a fusion of PSD-95 and mTurquoise [85] flanked by of AgeI (5’) and EcoRI (3’) as detailed in [13], and this segment was inserted into FUGWm instead of EGFP using the AgeI and EcoRI sites. A point mutation was then inserted to convert mTurquoise into mTurquoise2 (Isoleucine to Phenylalanine; [40]). Sequencing confirmed 100% identity with Rattus norvegicus discs large homolog 4 (NM_019621.1). All cloning and gene synthesis was done by Genscript (Piscataway NJ, US). The construct used to express EGFP:SynI (FU-Syn:EGFP-W) was provided as a generous gift by Craig C. Garner [86].

GCaMP6s [41] was expressed using an Adeno Associated Viral (AAV) vector obtained from the Penn Vector Core (University of Pennsylvania).

Lentiviral particles were produced in house as previously described [17]. Briefly, HEK293T cells were transfected using Lipofectamine 2000 (Invitrogen), a mixture of the three ViraPower kit packaging plasmids (Invitrogen), and the expression vector. Lentiviral stocks were prepared by collecting the supernatant after 48 h, filtering it using 0.45-μm filters, and storing it as small aliquots at -80°C.

Transduction of monolithic cortical cultures was performed at 5 d in vitro by adding 10–20 μl of lentiviral stock solution to each MEA dish. Transduction of modular cultures was performed as described above.

### Electrophysiological Recordings

MEA network activity was recorded using a commercial 60-channel headstage (Inverted A1060, MCS). Signals were first amplified by the internal headstage amplifier (1024x), multiplexed into 16 channels, amplified further (x10) by a 16-channel amplifier (Alligator technologies, US), and then digitized by an A/D converter (Microstar Laboratories, US) at 12 KSamples/sec per channel. Software used for data acquisition and display was based on AlphaMap (Alpha-Omega, Israel). Spiking activity data were stored as threshold crossing events (threshold = -40 μV) and analyzed offline using custom scripts written within the Matlab (MathWorks, US) programming environment.

### Long-Term Imaging

Fluorescence and brightfield images were acquired using a “homemade” confocal laser scanning microscope built around a Zeiss Axio Observer Z1. All imaging was carried out using a 40×, 1.3 N.A. Fluar objective (Zeiss). The system, controlled by software written by one of us (NEZ), allows for automated, multisite time-lapse microscopy. The MEA headstage described above was attached to the system’s motorized stage (Märzhäuser Wetzlar, Germany), and the MEA dishes were placed firmly within it.

PSD-95:mTurq2 and Cer:SV2 were excited using a 457-nm solid state laser (Cobolt, Sweden). PSD-95:EGFP, EGFP:SynI, and GCaMP6s were excited using a 488-nm solid state laser (Coherent, US). Fluorescence emissions were filtered through 467–493-nm and 500–550-nm bandpass filters (Semrock, US and Chroma Technology, US). Laser intensity modulation of the 457-nm solid state laser in experiments such as those described in Fig 7 was performed using the digital interface and software provided by the manufacturer.

Time lapse recordings were typically performed by averaging five frames collected at 10–11 focal planes (0.9 μm apart). Images were collected at a resolution of 640 x 480 pixels, 12 bits/pixel. The confocal aperture was kept fully open to minimize illumination intensities. The software-controlled motorized stage was used to collect data sequentially from up to 12 predefined locations. PSD-95:EGFP was imaged at 30–60-min intervals and Cer:SV2 at 7.5-h intervals. PSD-95:mTurq2 was imaged at 1-h intervals and EGFP:SynI at 1–3-h intervals. Focal drift was corrected before collecting data from each location by automatically locating the glass/medium interface plane and moving the focal position to a user-defined offset above this plane.

GCaMP6s transduced axons were imaged for ~1 min (600 frames, ~130 msec per frame) using a cooled EMCCD (Andor) controlled by custom written software.

### Environmental Conditions During Long-Term Experiments

To maintain neuronal network viability, the MEA dishes were covered with a “cap” equipped with ports through which sterile air mixtures and perfusion media were introduced and removed [6,17,24]. In addition, the cap was equipped with a dipping reference electrode made of thin platinum wire and a removable transparent glass window. The preparations were continuously perfused with feeding media at a rate of 2 ml/day using silicone tubes connected to the cap through the aforementioned ports and an ultra-slow peristaltic pump (Instech Laboratories Inc., US). In addition, a 95% air/5% CO_2_ sterile mixture was streamed continuously into the dish at rates regulated by a high-precision flow meter (Gilmont Instruments, US). The MEA dishes were heated to 36–37°C by the heating base at the bottom of the headstage/amplifier and by a custom objective heater as previously described [17].

### Pharmacological Manipulations

To minimize perturbations, all pharmacological agents were added to 100 μl media drawn from the MEA dish by temporarily removing the aforementioned caps glass window. The media was then returned and mixed gently using a sterile pipette, followed by returning the removable glass window. The same reagents were then added to the perfusion media at identical final concentrations, which were 1 μM for TTX; (Alomone Labs) and 20 μM for CCh (Sigma).

### Imaging Data Analysis

Analysis of imaging data was performed using an application (“OpenView”) written by one of us (NEZ). This application provides features for automated tracking of punctate fluorescent spots in time series of multiple images and the quantification of their fluorescence over time (see [24] for further details). 9 × 9 pixel (~1.3 x 1.3 μm) regions of interest (“boxes”) were centered on postsynaptic puncta, and average pixel intensities within these boxes were obtained from maximal intensity projections of all focal (Z) sections. As the reliability of automatic tracking was not absolutely perfect, all tracking was verified and, whenever necessary, corrected manually. Puncta for which tracking was ambiguous were excluded. Concomitant juxtaposition of marked presynaptic puncta was verified at every relevant time point and Z section. Whenever presynaptic puncta disappeared (even for a single time point) or became separated from their putative postsynaptic counterpart (both in XY or Z plane), the data for this synapse were excluded. Identification of CI synapses as such was limited to short, relatively straight axonal stretches, which did not intersect with other axons within the short stretch. To further facilitate CI disambiguation, low-magnification images of the imaged areas were collected for the purpose of resolving the branching structure of labeled axons and determining if axonal segments could be traced back to common origins. By keeping axonal labeling as sparse as possible, these procedures allowed for high-confidence CI synapse identification. Analysis of GCaMP6 time series was performed by first averaging four frames obtained between network bursts and thereafter subtracting these images from all images in the time series. GCaMP6 fluorescence was then quantified using OpenView as described above.

Covariance of CI synapses was calculated after smoothing PSD-95:EGFP or PSD-95:mTurq2 data with a 2.5- to 3-h low-pass filter (depending on imaging frequency). For CI synapse pairs (Figs 3 and 4 and 6D–6F, S4D–S4F, S5C and S5D and S6C and S6D Figs), covariance of CI synapses was calculated for the two synapses belonging to each pair, whereas covariance for non-CI synapses was calculated for CI_1_ to Ref_1_, CI_2_ to Ref_2_, CI_1_ to Ref_2_, and CI_2_ to Ref_1_ (four comparisons) to minimize potential effects of inter-synaptic distance. For multiple CI synapses formed between one axon and any dendrite (Fig 6A–6C, S4A–S4C, S5A and S5B and S6A and S6B Figs), covariance values for all possible CI pairs and CI-Ref pairs were calculated. Pearson’s and Spearman’s covariance values were calculated using Matlab and Microsoft Excel (using the Real Statistics Resource Pack; http://www.real-statistics.com). Data compilation, statistical testing, and plotting were performed using Microsoft Excel (and Real Statistics). Image examples (Figs 2, 5 and 7 and S2 Fig) were prepared using OpenView and Adobe Photoshop. Final figures were prepared using Microsoft PowerPoint.

### Quantifying the Coupling of Activity in Modular Networks

Sampled raw activity measurements were analyzed using custom written scripts in Matlab. Briefly, specific algorithms were used to identify bursting activity in each module (defined as activity in at least 25% of active electrodes in the module during 300-msec windows). A successful propagation of a burst from one module to the other was defined as a burst initiated in one of the modules followed by the appearance of a burst in the second module with a delay of no more than 50 msec between first spikes in each burst. The inter-modular synchronization measure *S* was calculated as

$$S=\;(\frac{B_{j}}{B_{1}+B_{2}+B_{j}})$$

where *B*_*j*_ is the number of joint bursts, and *B*_*1*_ and *B*_*2*_ are the number of bursts in modules #1 and #2, respectively, which did not propagate into the second module.

## Supporting Information

S1 Data Data used for the generation of main figures. (XLSX)

S1 Fig Size remodeling covariance of CI and non-CI synapses in monolithic networks—analyses based on Spearman’s rank correlation. (A) Distributions of size remodeling covariance values for CI and non-CI synapse pairs (92 CI pairs from 24 neurons in 6 experiments). Inset: Same data shown as cumulative histogram. (B,C) Average (±SEM) size remodeling covariance for all CI and non-CI synapse pairs in spontaneously active networks (B) and after suppressing spontaneous activity with TTX (C). (D,E) Same as (B,C)—data pooled by experiment. Statistical significance values based on two-tailed Mann-Whitney *U* tests. Source data provided in S1 Data.
(PDF)

S2 Fig Effects of TTX and Carbachol on average synapse size. (A) Changes in average PSD-95:EGFP fluorescence over a period of 24 h before and after the suppression of spontaneous network activity with TTX (213 synapses from 12 neurons from 3 experiments). (B) Changes in average PSD-95:EGFP fluorescence over a period of 15 h before and 12 h after exposure to Carbachol (20 μM; 194 synapses from 8 neurons from 2 experiments).
(PDF)

S3 Fig Ca^2+^ Imaging of presynaptic boutons of neurons originating in presynaptic module. (A) Presynaptic boutons of neurons expressing GCaMP6s. Maximum intensity image of 600 frames obtained at ~7 frames/sec. Background (created by averaging four frames obtained between bursts) was subtracted from image. (B) Same image as in (A), showing analysis regions of interest (ROI) placed over 17 boutons. (C) Correlation (Pearson’s) of GCaMP6s fluorescence profiles measured for each bouton, with the fluorescence profile of the bouton marked with asterisk, color coded according to color scale at the bottom of the panel. (D) Fluorescence profiles of three boutons labeled in panels A–C. An excellent correlation is observed between the fluorescence profiles, although, occasionally, slight differences are detectable (arrow). This might indicate that the boutons shown here belong to two axons (compare with color coded correlation in [C]); yet, the very high correlation values suggest that the activity histories of such axons are nevertheless very similar. (E) Comparison of Ca^2+^ transients averaged for all 17 boutons in this field of view with network activities (sum of all action potentials in 100-msec bins) recorded from MEA electrodes in the pre- and postsynaptic modules. Note the near-perfect correspondence with network activity recorded in the presynaptic module and the poor correspondence with network activity recorded in the postsynaptic module, confirming that the activities conveyed by axons traversing the barrier reflect the activities of presynaptic module neurons.
(PDF)

S4 Fig Size remodeling covariance of CI and non-CI synapses in modular networks—analyses based on Spearman’s rank correlation. (A) Distributions of size remodeling covariance values for all CI and non-CI synapse pairs (271 CI pairs from 29 neurons from 8 experiments). Inset: Same data shown as cumulative histogram. (B) Average (±SEM) size remodeling covariance for all CI and non-CI synapse pairs. (C) Same as (B)—data pooled by experiment. (D) Distributions of size remodeling covariance values for all CI_SD_ (that is, same axon, same dendrite) and non-CI synapse pairs (91 CI_SD_ pairs from 29 neurons from 8 experiments). Inset: Same data shown as cumulative histogram. (E) Average (±SEM) size remodeling covariance for all CI_SD_ and non-CI synapse pairs. (F) Same as (E)—data pooled by experiment. Statistical significance values based on two-tailed Mann-Whitney *U* tests. Source data provided in S1 Data.
(PDF)

S5 Fig Size remodeling covariance of CI and non-CI synapses in modular networks (high stringency data set, Pearson’s correlation). Same data as in Fig 6, but for subsets of the most stringently selected CI synapses (exclusion of relatively dim puncta; see main text for further details). (A) Distributions of size remodeling covariance values for all CI and non-CI synapse pairs (103 CI pairs from 29 neurons from 8 experiments). Inset: Same data shown as cumulative histogram. (B) Average (±SEM) size remodeling covariance for all CI and non-CI synapse pairs. (C) Distributions of size remodeling covariance values for all CI_SD_ (that is, same axon, same dendrite) and non-CI synapse pairs (40 CI_SD_ pairs from 29 neurons from 8 experiments). Inset: Same data shown as cumulative histogram. (D) Average (±SEM) size remodeling covariance for all CI_SD_ and non-CI synapse pairs. Statistical significance values based on two-tailed Mann-Whitney *U* tests. Source data provided in S1 Data.
(PDF)

S6 Fig Size remodeling covariance of CI and non-CI synapses in modular networks (high stringency data set, Spearman’s correlation). Same data as in Fig 6, but for subsets of the most stringently selected CI synapses (exclusion of relatively dim puncta; see main text for further details). (A) Distributions of size remodeling covariance values for all CI and non-CI synapse pairs (103 CI pairs from 29 neurons from 8 experiments). Inset: Same data shown as cumulative histogram. (B) Average (±SEM) size remodeling covariance for all CI and non-CI synapse pairs. (C) Distributions of size remodeling covariance values for all CI_SD_ (that is, same axon, same dendrite) and non-CI synapse pairs (40 CI_SD_ pairs from 29 neurons from 8 experiments). Inset: Same data shown as cumulative histogram. (D) Average (±SEM) size remodeling covariance for all CI_SD_ and non-CI synapse pairs. Statistical significance values based on two-tailed Mann-Whitney *U* tests. Source data provided in S1 Data.
(PDF)

## Abbreviations

4Q: four-quadrant
AMPA: α-amino-3-hydroxy-5-methyl-4-isoxazolepropionic acid
CI: commonly innervated
CI_SD_: Commonly Innervated Same Dendrite
EGFP: enhanced green fluorescent protein
EMCCD: electron-multiplying charged couple device
MEA: multielectrode array
NMDA: N-methyl-D-aspartate
ρ: Spearman’s rank correlation coefficient
PDMS: polydimethylsiloxane
PSD: postsynaptic density
r: Pearson’s correlation coefficient
Ref: reference
TTX: tetrodotoxin
