# Supplementary material for: Relative Contributions of Specific Activity Histories and Spontaneous Processes to Size Remodeling of Glutamatergic Synapses
Source: PLoS Biol. 2016 Oct 24;14(10):e1002572. doi: 10.1371/journal.pbio.1002572 (PMC5077109; doi:10.1371/journal.pbio.1002572)
Supplement: S1 Fig — (A) Distributions of size remodeling covariance values for CI and non-CI synapse pairs (92 CI pairs from 24 neurons in 6 experiments). Inset: Same data shown as cumulative histogram. (B,C) Average (±SEM) size remodeling covariance for all CI and non-CI synapse pairs in spontaneously active networks (B) and after suppressing spontaneous activity with TTX (C). (D,E) Same as (B,C)—data pooled by experiment. Statistical significance values based on two-tailed Mann-Whitney U tests. Source data provided in S1 Data. (PDF) [file pbio.1002572.s002.pdf]

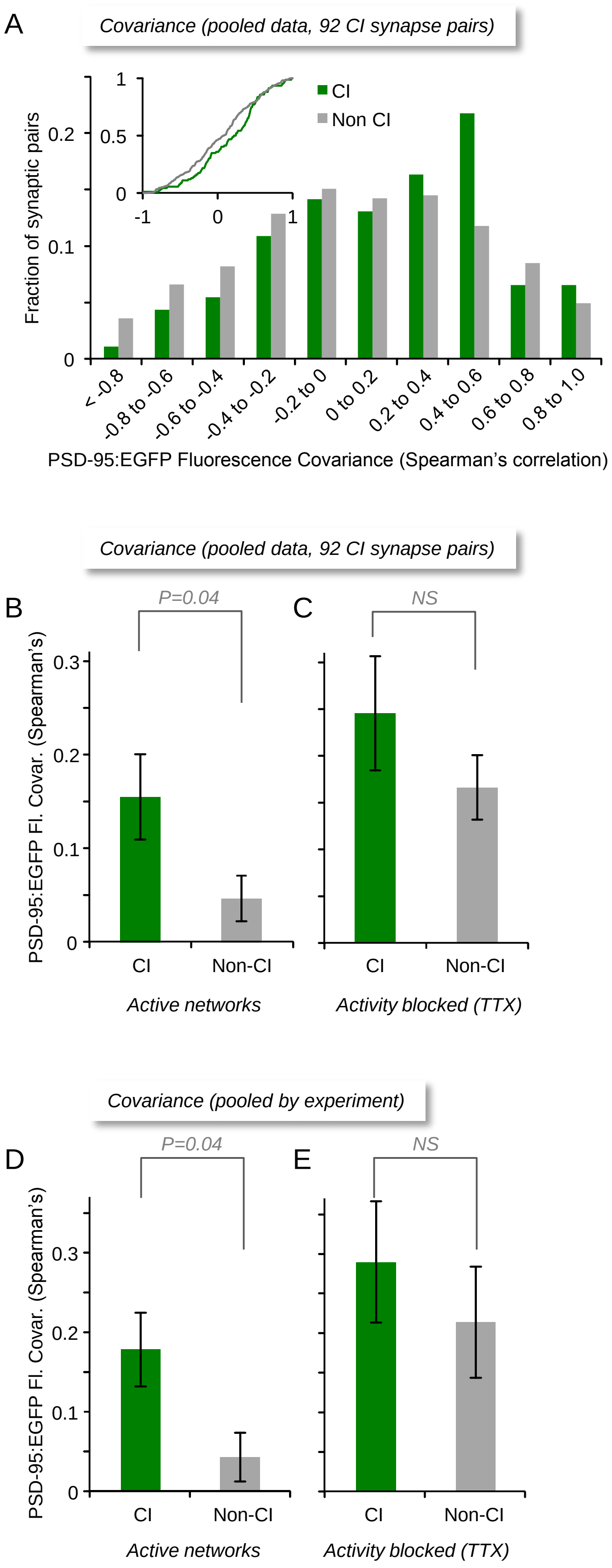


**S1 Fig:** Size remodeling covariance of CI and non-CI synapses in monolithic networks based on Spearman's rank correlation
