# Supplementary figures and images for: Relative Contributions of Specific Activity Histories and Spontaneous Processes to Size Remodeling of Glutamatergic Synapses
Source: PLoS Biol. 2016 Oct 24;14(10):e1002572. doi: 10.1371/journal.pbio.1002572 (PMC5077109; doi:10.1371/journal.pbio.1002572)

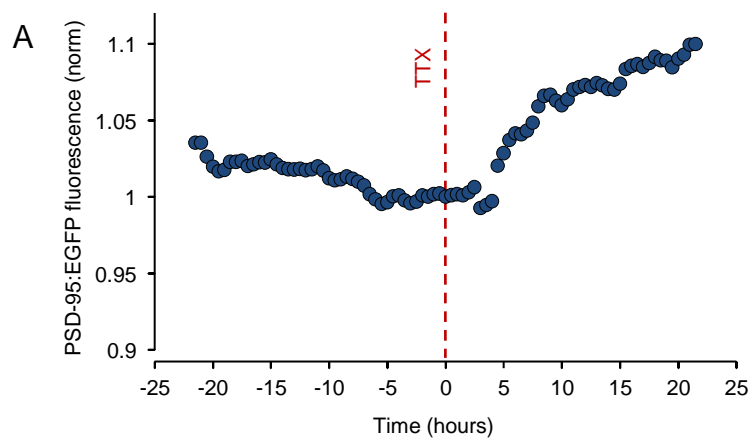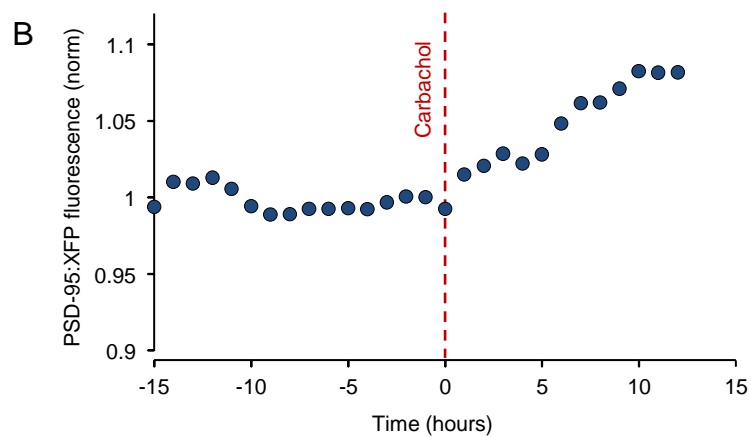

**S2 Fig:** Effects of TTX and Carbachol on average synapse size

Supplement: S2 Fig — (A) Changes in average PSD-95:EGFP fluorescence over a period of 24 h before and after the suppression of spontaneous network activity with TTX (213 synapses from 12 neurons from 3 experiments). (B) Changes in average PSD-95:EGFP fluorescence over a period of 15 h before and 12 h after exposure to Carbachol (20 μM; 194 synapses from 8 neurons from 2 experiments). (PDF) [file pbio.1002572.s003.pdf]

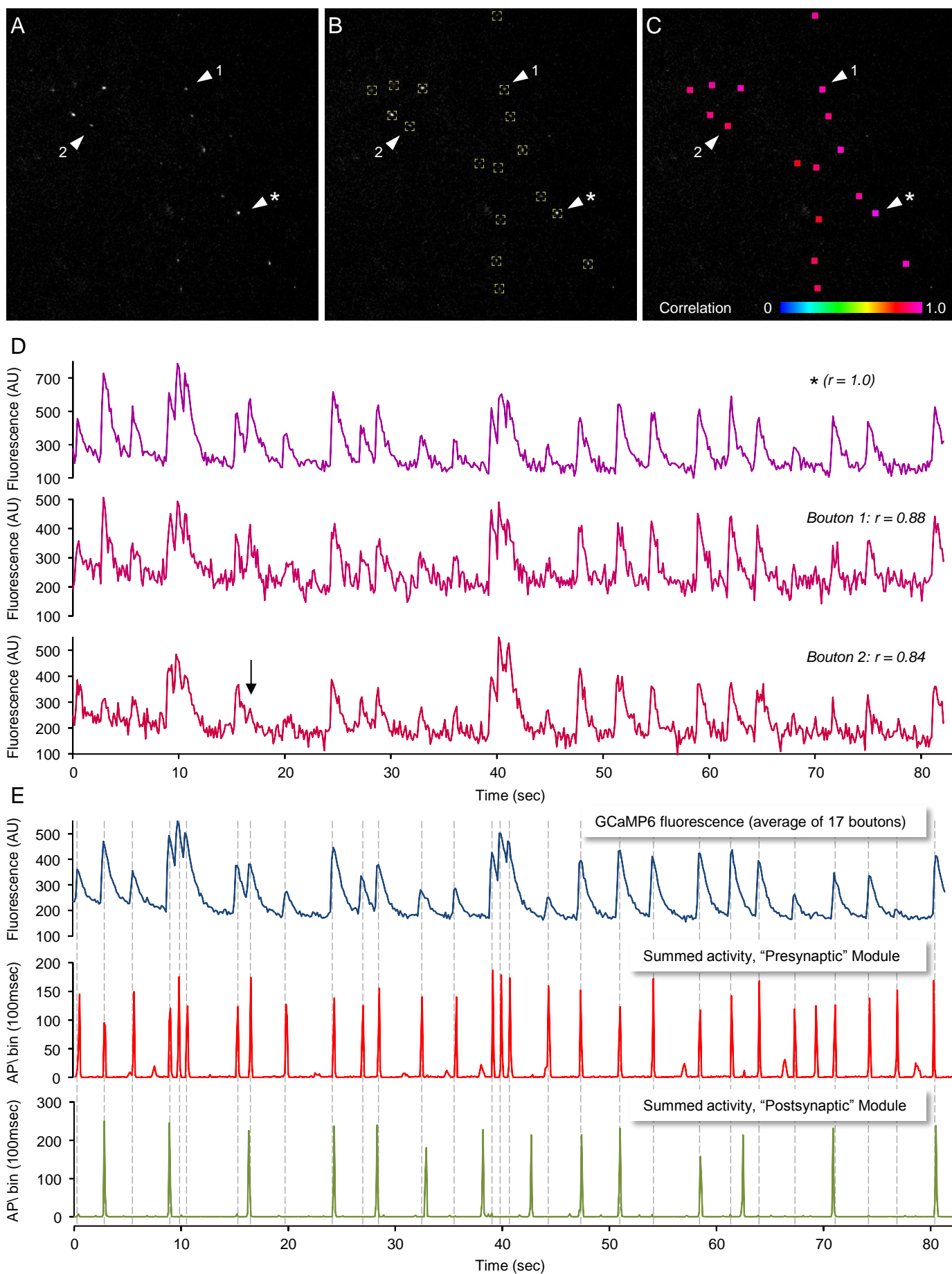

**S3 Fig:**  $\text{Ca}^{2+}$  Imaging of presynaptic boutons of neurons originating in presynaptic module

Supplement: S3 Fig — (A) Presynaptic boutons of neurons expressing GCaMP6s. Maximum intensity image of 600 frames obtained at ~7 frames/sec. Background (created by averaging four frames obtained between bursts) was subtracted from image. (B) Same image as in (A), showing analysis regions of interest (ROI) placed over 17 boutons. (C) Correlation (Pearson’s) of GCaMP6s fluorescence profiles measured for each bouton, with the fluorescence profile of the bouton marked with asterisk, color coded according to color scale at the bottom of the panel. (D) Fluorescence profiles of three boutons labeled in panels A–C. An excellent correlation is observed between the fluorescence profiles, although, occasionally, slight differences are detectable (arrow). This might indicate that the boutons shown here belong to two axons (compare with color coded correlation in [C]); yet, the very high correlation values suggest that the activity histories of such axons are nevertheless very similar. (E) Comparison of Ca2+ transients averaged for all 17 boutons in this field of view with network activities (sum of all action potentials in 100-msec bins) recorded from MEA electrodes in the pre- and postsynaptic modules. Note the near-perfect correspondence with network activity recorded in the presynaptic module and the poor correspondence with network activity recorded in the postsynaptic module, confirming that the activities conveyed by axons traversing the barrier reflect the activities of presynaptic module neurons. (PDF) [file pbio.1002572.s004.pdf]
