# Supplementary material for: Relative Contributions of Specific Activity Histories and Spontaneous Processes to Size Remodeling of Glutamatergic Synapses
Source: PLoS Biol. 2016 Oct 24;14(10):e1002572. doi: 10.1371/journal.pbio.1002572 (PMC5077109; doi:10.1371/journal.pbio.1002572)
Supplement: S5 Fig — Same data as in Fig 6, but for subsets of the most stringently selected CI synapses (exclusion of relatively dim puncta; see main text for further details). (A) Distributions of size remodeling covariance values for all CI and non-CI synapse pairs (103 CI pairs from 29 neurons from 8 experiments). Inset: Same data shown as cumulative histogram. (B) Average (±SEM) size remodeling covariance for all CI and non-CI synapse pairs. (C) Distributions of size remodeling covariance values for all CISD (that is, same axon, same dendrite) and non-CI synapse pairs (40 CISD pairs from 29 neurons from 8 experiments). Inset: Same data shown as cumulative histogram. (D) Average (±SEM) size remodeling covariance for all CISD and non-CI synapse pairs. Statistical significance values based on two-tailed Mann-Whitney U tests. Source data provided in S1 Data. (PDF) [file pbio.1002572.s006.pdf]

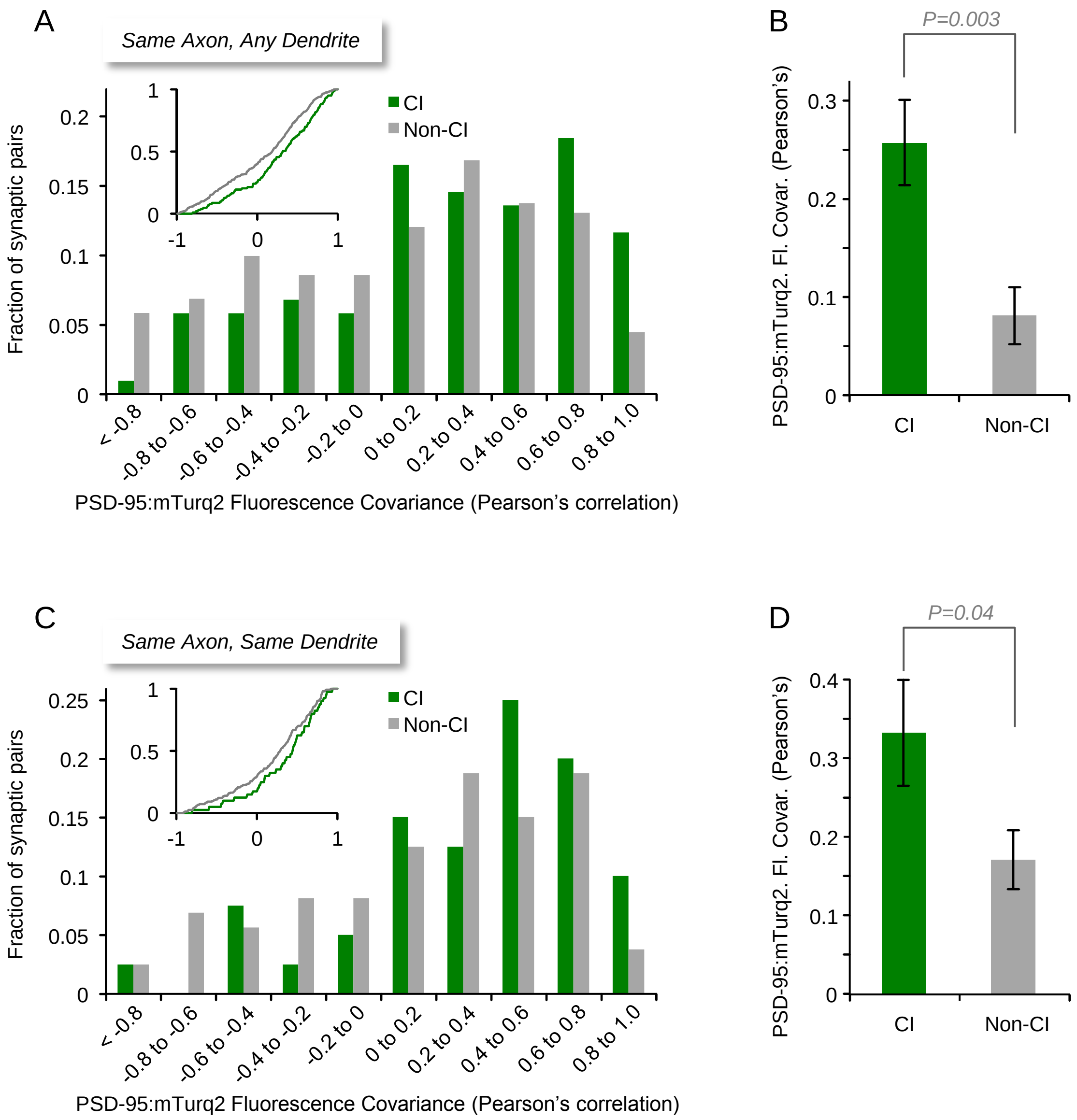


**S5 Fig:** Size remodeling covariance of CI and non-CI synapses in modular networks (high stringency data set, Pearson's correlation)
